# Carotenoid pattern intake and relation to metabolic status, risk and syndrome, and its components – divergent findings from the ORISCAV-LUX-2 survey

**DOI:** 10.1017/S0007114524000758

**Published:** 2024-07-14

**Authors:** Jaouad Bouayed, Farhad Vahid

**Affiliations:** 1 Université de Lorraine, LCOMS/Neurotoxicologie Alimentaire et Bioactivité, 57000 Metz, France; 2 Nutrition and Health Research Group, Department of Precision Health, Luxembourg Institute of Health, Strassen, Luxembourg

**Keywords:** Central obesity, Dietary patterns, Hyperglycaemia, Hypertension, Dyslipidemia, Cardiometabolic health

## Abstract

Carotenoids are generally associated with health-beneficial effects; however, their intake patterns related to the metabolic syndrome (MetS) and its components remain controversial. This cross-sectional study investigated associations between dietary intakes of individual carotenoids, fruits and vegetables, and the MetS and its components. Dietary intakes of 1346 participants of the Observation des Risques et de la Santé Cardio-Vasculaire au Luxembourg (ORISCAV-LUX-2) study were investigated by a 174-item FFQ, and carotenoid intake was determined by linking findings using mainly the USDA food databases. Components of MetS and complementary variables, including anthropometric (BMI, waist circumferences and waist:hip ratio) and biological parameters (TAG, HDL-cholesterol, fasting blood glucose and blood pressure), were measured. Logistic (for MetS) and linear multivariable regression models (including assessing MetS as scores) adjusted for various confounders were created. *α*-and *β*-Carotene, as well as lutein + zeaxanthin, were inversely associated with MetS (also when it was measured on a continuous scale), reducing the odds for MetS by up to 48 %. However, lycopene, phytoene and phytofluene were rather positively associated with MetS scores and its components, though these adverse effects disappeared, at least for lycopene, when controlling for intakes of tomato-based convenience foods, in line with indicating a rather unhealthy/westernised diet. All these associations remained significant when including fruits and vegetables as confounders, suggesting that carotenoids were related to MetS independently from effects within fruits and vegetables. Thus, a high intake of carotenoids was bidirectionally associated with MetS, its severity, risk and its components, depending on the type of carotenoid. Future investigations are warranted to explore the inverse role that tomato-based carotenoids appear to suggest in relation to the MetS.

## Graphical abstract

**Figure:**
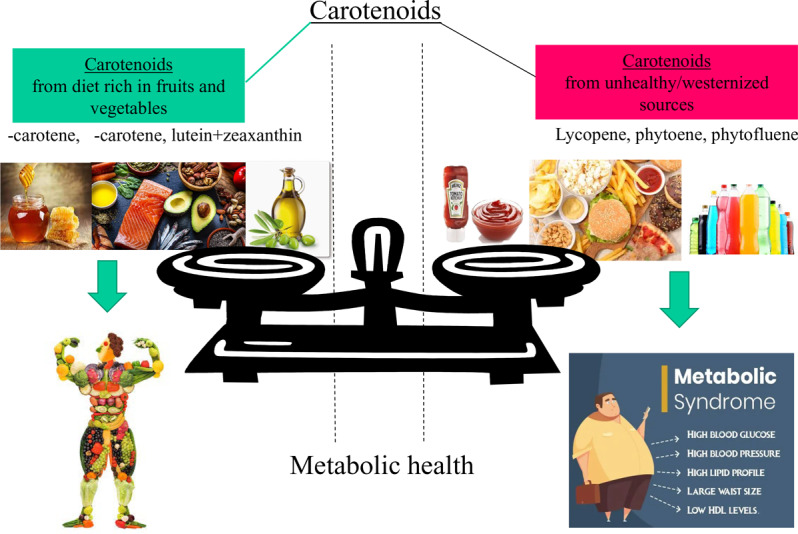

Almost 2400 years ago, Hippocrates, the father of modern medicine, led the way by his famous aphorism ‘Let food be thy medicine and medicine by thy food’. Epidemiological evidence has shown that healthy dietary habits aid in maintaining good health; however, unhealthy habits have been related to several multifactorial non-communicable diseases^(1–4)^. The metabolic syndrome (MetS) corresponds to an early pathological condition that increases the risk of developing a plethora of chronic non-communicable diseases such as type 2 diabetes, CVD, including stroke^(5–7)^, Alzheimer’s disease^(8)^ and cancer^(9)^. It affects approximately a quarter of the adult population worldwide^(10)^ and also in developed countries. In Luxembourg, the prevalence is about 25 %^(11)^.

MetS is a constellation of several cardiometabolic risk factors and is diagnosed when three out of the five following conditions are met: (i) abdominal obesity, (ii) elevated blood pressure, (iii) elevated fasting blood glucose (FBG), (iv) reduced level of ‘good’ cholesterol (HDL-cholesterol) and (v) high levels of triglycerides (TAG)^(6,7,9,12)^. It is estimated that the coexistence of all these factors increases the risk of type 2 diabetes by five times^(13)^, a disease that affects 500 million people worldwide, including 60 million in Europe^(14)^. In addition, it is estimated that the clustering of the set of MetS components increases by 1·7 the risk of CVD^(13)^, leading to an annual death toll worldwide of about 18 million individuals^(14)^. It is important to emphasise that other metabolic abnormalities characterise MetS, such as oxidative stress and chronic low-grade inflammation, even if they are not necessarily part of the traditional diagnostic criteria of this syndrome^(8,14)^. Many researchers have though recommend the introduction of C-reactive protein, a marker of inflammation, into the diagnostic criteria for MetS, and oxidative stress markers, such as measured by F2-isoprostanes, are also frequently recommended^(14,15)^.

The prevalence of MetS and its cardiometabolic risk factors can be reduced by fostering a healthy lifestyle with increased physical activity and healthy dietary practices^(1,2,7)^. For instance, two meta-analyses found an inverse association between fruit and/or vegetable consumption and MetS risk^(16,17)^. Health beneficial effects of diets rich in fruits and vegetables on MetS are attributed due to their low energy content, low total carbohydrate to fibre ratios, and also their bioactive constituents, including dietary fibre, vitamins, minerals, polyphenols and also carotenoids^(1,2,13)^.

Carotenoids are the most abundant secondary plant metabolites in the human body^(18,19)^. Though the consumption of carotenoids is relatively low, for example, it is 100 times lower than that of polyphenols, the total concentration of native carotenoids in plasma is about 0·9 to 2·5 µM, much higher than that of native polyphenols, which is typically in the nanomolar range^(19)^. Total blood carotenoids have been proposed as a cardiometabolic health index, with concentrations < 1 µM highly increasing the risk for cardiometabolic diseases^(20)^. The optimum recommended concentrations of *β*-carotene to reduce the risk of ischaemic heart disease were set at > 0·4 µM^(21)^. In a recent cross-sectional study, it has been shown that plasma *β*-carotene levels decreased with increasing MetS severity in adult Polish with MetS^(22)^. Total carotenoid concentrations were also found significantly lower in adults with five MetS components^(22)^. In this regard, a recent meta-analysis related low circulating carotenoid levels to MetS with lipid disturbances^(23)^. In another recent meta-analysis, it was demonstrated that people with high levels of circulating carotenoids were significantly more protected from MetS compared with those with low levels^(13)^. In addition to the inverse association between serum total carotenoids and MetS, the meta-analysis also revealed an inverse association between several individual carotenoids and MetS. For instance, this association was stronger for *β*-carotene than for *α*-carotene, followed by *β*-cryptoxanthin^(13)^.

Evidence suggests that regarding MetS risk factors, carotenoids may play critical but differential roles in adipose tissue biology, including the control of adipogenesis, oxidative stress, and the production of adipokines and inflammatory mediators that affect central adiposity distribution and the occurrence of insulin resistance^(13)^. Indeed, this is exactly what has been proposed in previous reviews^(24,25)^ due to the involvement of especially PPAR receptors and adipogenesis, with PPAR being targeted by carotenoids and their apocarotenoid metabolites^(26)^. Other mechanistic pathways via which carotenoids may be implicated in the aetiology of MetS and its components include direct antioxidant effects^(18,27)^, their possible positive effects on the gut microbiota^(20,28)^, and their interactions with transcription factors such as NF-kB and Nrf2 as well as additional nuclear receptors, namely RAR/RXR^(29)^.

Carotenoids have been, in several epidemiological studies, considered as valuable indicators of a diet rich in fruits and vegetables^(30–33)^, being a characteristic of, for example, traditional Mediterranean diets, supplying also secondary plant metabolites at physiological ranges^(27,34)^. For example, elevated levels of serum lycopene or intake including lycopene-rich tomatoes have been regarded as protective against MetS risk^(23,35–37)^. On the contrary, a high intake of lycopene from processed tomato-based products has also been regarded as an indicator of a westernised diet^(20)^, which has been positively associated with MetS^(38,39)^. Therefore, the role of especially lycopene in the diet and its association with MetS remains somewhat unclear.

In this study, we aimed to assess the possible associations of carotenoid dietary intake as well as their profiles with the MetS, its scores, its cardiometabolic components and anthropometric characteristics by using data from Luxembourg adults participating in the Observation des Risques et de la Santé Cardio-Vasculaire au Luxembourg (ORISCAV-LUX-2) study. More specifically, we hypothesised that the intake of lycopene and possibly other tomato-based carotenoids, due to their further association with westernised dietary patterns, may be differentially related to MetS compared with the intake of other carotenoids that rather characterise a healthy diet rich in fruits and vegetables.

## Materials and methods

### ORISCAV-LUX-2 study

**Fig. 1.**
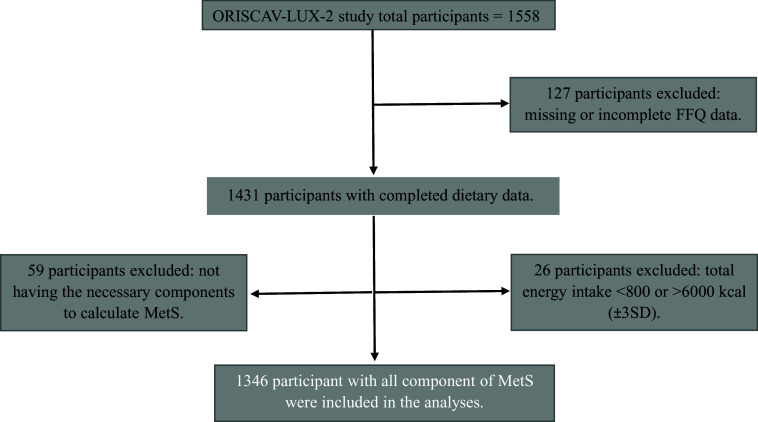
Flow chart of participants’ sample progression. ORISCAV-LUX, Observation des Risques et de la Santé Cardio-Vasculaire au Luxembourg; MetS, metabolic syndrome.

In 2016–2017, an observational study, termed ‘Observation des Risques et de la Santé Cardio-Vasculaire au Luxembourg’ (ORISCAV-LUX-2), was carried out in Luxembourg^(40)^ as a second wave to the previously conducted ORISCAV-LUX study in 2006/2007^(41)^. A detailed description of the ORISCAV-LUX-2 study has been reported previously^(40,42,43)^. The flow chart of the participants’ sample progression is shown in Fig. 1. From all participants (*n* 1558) selected following sociodemographic criteria, including the district of residence, age and sex, 212 individuals with incomplete data, including FFQ and metabolic data, were removed from the present study (Fig. 1). Thus, 1346 participants (25–79 years) were retained in this cross-sectional study. Volunteers self-reported their lifestyle habits, including diet, drinking, physical activity and smoking, as well as sociodemographic aspects and health conditions such as job, income, marital status and medical history. Anthropometric measurements, including BMI and waist and hip circumference, were collected by trained nurses. BMI was estimated as weight in kg divided by the square of height in metres (kg/m^2^). Waist:hip ratio (WHR) was calculated by dividing the circumference of the waist by the circumference of the hip. Biological samples, including blood, were stored under appropriate conditions in the integrated BioBank of Luxembourg. Fasting blood tests were carried out to analyse the following markers: TAG, HDL-cholesterol and FBG. The commercial accredited company Ketterthill (Esch-Sur-Alzette) conducted medical analyses. The National Research Ethics Committee (CNER, No 201-505/12) and the National Commission for Private Data Protection (CNPD) approved the study’s design and the information collected.

### FFQ

The long-term habitual dietary intake of adult residents in Luxembourg was assessed employing a validated semi-quantitative 174-item FFQ, capturing food intake over the preceding 3 months^(42)^. The FFQ was translated into the four frequently used languages in Luxembourg, namely French, English, German and Portuguese; self-reported food intake by adult residents was then back-translated into French to ensure linguistic validity. The FFQ consisted of nine food categories, namely vegetables (cooked and raw), fruits (including canned fruits, compotes, salted and unsalted dried fruits, fresh fruit juices, and fruit juices), meat-poultry-fish-egg, prepared dishes, dairy products, fats, starchy foods, miscellaneous and drinks, including total 174 food and beverage items. Trained nurses guided the respondents to fill out the auto-administered questionnaires and reviewed the correctness of their responses. Weekly and monthly frequency intakes were transformed into daily equivalent frequency intakes. The daily intake of each food (g/d) and drink (ml/d) item for each participant was obtained by multiplying the intake frequency (daily equivalents) by the portion size.

### Carotenoid intake assessment

The carotenoids most frequently consumed within the Luxembourgish diet, including *β*-carotene, *α*-carotene and *β*-cryptoxanthin (provitamin A species), and lycopene, lutein + zeaxanthin, astaxanthin, phytoene, phtofluene, neoxanthin and violaxanthin (non-provitamin A species), were assessed, by linking the FFQ data to several carotenoid databases. The concentrations (µg/100 g food item) of provitamin A species, lycopene, and lutein + zeaxanthin in food and drink items contained in the FFQ were extracted using the US Department of Agriculture (USDA) database (website: https://fdc.nal.usda.gov/, accessed in 2023) and the USDA-NCC Carotenoid Database^(44)^. As the other carotenoids were not included in the USDA database, additional sources were employed to estimate their intake: Concentrations of phytoene, phytofluene, neoxanthin and violaxanthin were estimated using a local database^(19)^. Concentration of astaxanthin in fish and seafood items was estimated using the EFSA database^(45)^. Carotenoid intake (µg/d) was then computed by multiplying food intake (g/d) by carotenoid concentration (µg/100 g). Afterwards, the participant’s total daily intake for each individual carotenoid, provitamin A and non-provitamin A carotenoids, colourless carotenoids (phytoene + phtofluene), epoxycarotenoids (neoxanthin + violaxanthin), and the sum of all carotenoids consumed by each participant was obtained.

### Metabolic syndrome criteria and calculation of its continuous scores

The dichotomous diagnosis of MetS in participants was established following the definition of the National Cholesterol Education Program – Adult Treatment Panel III (NCEP-ATP III)^(7,11)^. Thus, the occurrence of three or more of the following cardiometabolic components were required for MetS diagnosis: (i) waist circumference: ≥ 102 cm for men and ≥ 89 cm for women; (ii) high TAG level: ≥ 150 mg/dl (1·7 mmol/l); (iii) reduced HDL-cholesterol: < 40 mg/dl (1·04 mmol/l) in men and < 50 mg/dl (1·3 mmol/l) in women; (iv) increased blood pressure (systolic blood pressure (SBP) or diastolic blood pressure (DBP)): ≥ 130 or 85 mmHg, respectively; and (v) impaired FBG: ≥ 100 mg/dl (5·6 mmol/l), or components were considered positive if any of them were treated by drugs (except for waist circumference).

A validated continuous score of MetS (siMetS), reflecting the metabolic status of all participants score, was computed employing the following formula^(46,47)^ that includes the cut-off values from the diagnosis of MetS:

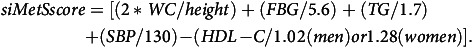




In addition, a validated continuous score of MetS, derived from the siMetS score, was used to quantify metabolic risk (siMetS risk score), that is, the risk of cardio/cerebrovascular events, in all ORISCAV-LUX-2 participants^(48)^. The calculation of the siMetS risk score relies on the age of participants, with an age of 45 years (for men) or 50 years (the average age of menopause) as a threshold for increased incidence of cardio/cerebrovascular events^(47)^. In addition, the presence of family antecedents of cardiovascular events heightens the risk score of MetS by 20 %^(47)^. siMetS risk score was computed employing the following formula^(47)^:

siMetS risk score = (siMetS score × (Age /45 (men) or 50 (women)) × 1·2 or 1 (if the presence or not of the family antecedent of a cardiovascular event, respectively)).

The units of continuous variables used to calculate siMetS score were mmHg for SBP and DBP, centimetre (cm) for WC (waist circumference) and height, and mmol/l for FBG, TAG, and HDL-cholesterol. The siMetS and siMetS risk scores were calculated separately for women and men using different coefficients in the formulas.

### Statistical analysis

The normality of the data distribution and homogeneity of variance was estimated by using Q-Q normality plots and box plots. All variables (unless otherwise noted) were transformed to a logarithmic scale to obtain a normal distribution. Bivariate correlations between intakes of carotenoids were analysed with Spearman’s rho.

For the descriptive analyses, two independent group comparisons were made to study log-transformed continuous data using the independent-samples *t* tests. The *χ* ^2^ test was employed for comparing categorical variables.

Four regression models were set up in our study to investigate the association of carotenoids, fruits, and vegetables with MetS and its related variables. Linearity between independent and dependent continuous variables was verified by plotting dependent variables *v*. standardised residuals using regression scatter plots and correlation coefficients. The first model was an unadjusted one (crude model). The second model was adjusted for age and sex (model 2, except for the risk score of siMetS). In addition, a set of the identified confounders was retained based on the literature. In model 3, fruits and vegetables were considered as independent variables in addition to carotenoids, and thus the retained confounders included age, sex (men/women), marital status (four categories), current smoking status (four categories), job status (four categories), income (eight categories), birth country (four categories) and total energy intake. Participants with missing income and job status values were included in the category ‘did not answer’. The fourth model was the fully adjusted one used to investigate the role of carotenoids independently from fruit and vegetable intake on MetS (model 4 = model 3 plus adjusted for fruit and vegetable intake).

Multivariable logistic regression was carried out to investigate the association between MetS (as a categorical dependent variable) and the consumption of carotenoids, fruits, and vegetables (log-transformed explanatory variables) using the four models. Multivariable linear regression was further carried out to seek the associations between dietary intake of carotenoids, fruits, and vegetables (log-transformed predictor variables) and continuous dependent variables, including the scores of MetS as well as its components and the different measured anthropometric characteristics, using the four models. Finally, sensitivity analyses were carried out for lycopene, phytoene and phytofluene, in which tomato-based convenience food items rich in these carotenoids (ketchup, lasagna with meat and tomato sauce, pizzas, and burgers) were included as confounding factors.

For all statistical analyses, a *P*-value below 0·05 (two-sided) was considered significant, and a non-significant *P*-value above this value but below 0·1 was considered tendency. The raw data were reported as median and interquartile ranges, and the categorical variables were reported as numbers and percentages. All statistical analyses were performed using SPSS 25.0 (SPSS Inc. IBM).

## Results

### Descriptive analysis (non-adjusted findings)

In the ORISCAV-LUX-2 survey, the overall percentage of MetS within the included individuals was 27·1 %. However, a significantly higher percentage was found for men (32·8 %) in comparison with women (22·1 %) (*P* < 0·001) (Table 1). Moreover, both continuous scores related to MetS (siMetS and siMetS risk scores) were significantly higher in men than women (*P* < 0·001). While the level of HDL-cholesterol was significantly higher in women compared with men (*P* < 0·001), all other MetS components, which were rather positively associated with MetS, were significantly elevated in men *v.* women (all *P* < 0·001). The same pattern was found when comparing MetS components between both men and women having MetS (all *P* < 0·001), except for FBG concentration (*P =* 0·109). Similarly, all MetS components were further found to be significantly different between both men and women without MetS (all *P* < 0·001). In this study, the number of women participants who had relatives (father, mother, brother or sister) having at least one cardiovascular event (myocardial infarction or stroke) was higher than men (Table 1).

**Table 1.** Distribution of the metabolic syndrome (MetS), metabolic outcomes and anthropometric characteristics in Luxembourgish participants (*n* 1346) of the ORISCAV-2 study. Scores of the MetS and MetS components are reported as median and interquartile ranges

Health outcomes	All participants				Men				Women			
	MetS [+]		MetS [−]		MetS [+]		MetS [−]		MetS [+]		MetS [−]	
MetS (number and %)	364·0	27·0 %	982·0	73·0 %	206·0	32·7 %	424·0	67·3 %	158·0	22·1 %	558·0	77·9 %
	1346·0 (100 %)				630·0 (46·8 %)				716·0 (53·2 %)			
siMetS score (metabolic status)	2·2 (0·9)				2·4 (0·9)				2·0 (0·8)			
	2·9	0·9	2·0	0·7	3·0	1·0	2·2	0·7	2·7	0·7	1·9	0·6
siMetS risk score (metabolic risk)	2·2 (1·5)				2·7 (1·7)				1·8 (1·1)			
	3·3	1·8	1·9	1·2	3·9	1·4	2·4	1·2	2·4	1·4*	1·7	0·9*
Waist circumference (cm)	88·5 (17·2)				94·0 (17·2)				83·5 (15·5)			
	100·1	17·7	85·2	14·9	105·2	16·1	91·0	12·8	94·2	16·1	81·1	13·8
Fasting blood glucose (FBG) (mg/dl)	89·0 (13·0)				92·0 (12·0)				87·0 (12·0)			
	98·0	15·0	87·0	10·0	99·0	15·5*	89·0	9·0	95·0	14·0*	85·0	10·0
HDL-cholesterol (mg/dl)	57·0 (19·0)				50·0 (15·0)				63·0 (17·0)			
	48·0	18·5	59·0	18·0	43·0	16·0	51·5	14·0	56·0	20·2	64·0	16·0
TAG (mg/dl)	88·0 (56·0)				101·0 (67·5)				79·0 (44·0)			
	116·0	82·0	80·0	45·0	142·0	95·0	93·0	50·7	99·5	63·7	73·0	39·0
Systolic blood pressure (SBP) (mmHg)	123·5 (22·5)				129·0 (20·0)				117·5 (22·5)			
	133·5	20·0	120·0	19·5	136·0	17·5	126·0	16·5	131·0	19·6	114·0	19·5
Diastolic blood pressure (DBP) (mmHg)	78·0 (14·0)				81·0 (13·5)				76·0 (14·5)			
	84·0	14·0	76·5	13·5	85·0	12·5	79·5	13·0	83·2	15·5	74·5	13·0
Number of participants with family history of cardio/cerebrovascular events	172·0 (100 %)				69·0 (40·1 %)				103·0 (59·9 %)			
	67·0	39·0 %	105·0	6·0 %	39·0	56·5 %	30·0	43·5 %	28·0	27·2 %	75·0	72·8 %

ORISCAV, Observation des Risques et de la Santé Cardio-Vasculaire au Luxembourg; NCEP-ATP III, National Cholesterol Education Program – Adult Treatment Panel III.

The MetS was diagnosed with the NCEP-ATP III criteria.

MetS [+] and MetS [−] account for the presence and absence of the MetS, respectively.

Distribution of MetS is reported as number and percentage (%).

siMetS score and siMetS risk score are continuous MetS scores used to quantify metabolic status and metabolic risk of cardio/cerebrovascular events, respectively.

*χ* ^2^ test was used to compare MetS numbers between groups.

Independent-samples *t* tests were used to compare between the metabolic outcomes of participants (log-transformed data).

All two group comparisons (in the same row) are significant (all *P* < 0·01), except for the two comparisons highlighted in the table with *P > 0·05).

Two group comparisons included all participants MetS [+] *v*. all participants MetS [−], men *v*. women, men MetS [+] *v*. women MetS [+] and men MetS [+] *v*. women MetS [+].

No statistical comparison was carried out for the number of participants with a history family of cardio/cerebrovascular events.

Family history of cardio/cerebrovascular events included father, mother, brother or sister having a cardiovascular event (myocardial infarction or stroke).

Daily intake of individual provitamin A species and the total sum of provitamin A species were significantly higher in women than men (all *P* < 0·05, Table 2). The same pattern was found for phytoene, phytofluene, phytoene + phytofluene, violaxanthin and epoxycartenoids (all *P* < 0·05). However, daily intake of lycopene (*P* < 0·001) and astaxanthin (*P* < 0·05) were significantly higher in men compared with women. Regarding the daily intake of neoxanthin, lutein + zeaxanthin, total non-provitamin A species and total carotenoids, no significant sex differences were found (all *P >* 0·05, Table 2).

**Table 2.** Daily intake of carotenoids (µg), fruits (g) and vegetables (g) reported as the median (interquartile range) of Luxembourgish participants (*n* 1346) of the ORISCAV-2 study

Dietary intakes	All participants (*n* 1346)					Men (*n* 630)					Women (*n* 716)					Men *v*. women	Men MetS [+] *v*. women MetS [+]	Men MetS [−] *v*. women MetS [−]
	MetS [+] (*n* 364)		MetS [−] (*n* 982)		*P*-value	MetS [+] (*n* 206)		MetS [−] (*n* 424)		*P*	MetS [+] (*n* 158)		MetS [−] (*n* 558)		*P*	*P*	*P*	*P*e
*α*-Carotene (µg/d)	2510 (3082)				***–***	2448 (3069)				–	2561 (3277)				–	***P =*** **0·027**	–	–
	2403	2966	2579	3130	***P =*** **0·072**	2358	2946	2584	3132	*P =* 0·232	2498	3088	2579	3321	*P =* 0·340	–	*P =* 0·237	*P =* 0·103
*β*-Carotene (µg/d)	6530 (6532)				–	6132 (6341)				–	6925 (7082)				–	***P =*** **0·008**	–	–
	6062	6429	6748	6563	*P =* 0·115	5741	6265	6360	6325	*P =* 0·175	6777	6805	6965	7122	*P =* 0·722	–	***P =*** **0·064**	***P =*** **0·080**
*β*-Cryptoxanthin (µg/d)	246 (273)				–	222 (263)				–	265 (289)				–	***P =*** **0·007**	–	–
	253	318	243	269	*P =* 0·384	239	291	210	269	*P =* 0·190	273	349	262	275	*P =* 0·720	–	*P =* 0·346	***P =*** **0·007**
Total provitamin A (µg/d)	9478 (9834)				–	8834 (9516)				–	9886 (10 131)				–	***P =*** **0·010**	–	–
	8755	9635	9750	9881	*P =* 0·105	8238	9603	9299	9436	*P =* 0·174	9589	9939	9988	10 611	*P =* 0·663	–	***P =*** **0·077**	***P =*** **0·086**
Lycopene (µg/d)	3565 (3543)				–	4220 (4337)				–	3197 (3218)				–	***P <*** **0·001**	–	–
	3568	3716	3563	3531	***P =*** **0·039**	3872	4357	4437	4369	***P =*** **0·010**	3197	3315	3197	3196	*P =* 0·189	–	***P =*** **0·065**	***P <*** **0·001**
Lutein + zeaxanthin (µg/d)	1632 (1890)				–	1628 (1778)				–	1635 (1946)				–	*P =* 0·463	–	–
	1612	1883	1641	1891	*P =* 0·929	1556	1658	1656	1810	*P =* 0·724	1666	2043	1632	1932	*P =* 0·922	–	*P =* 0·898	*P =* 0·425
Astaxanthin (µg/d)	47 (50)				–	50 (56)				–	45 (47)				–	***P =*** **0·048**	–	–
	47	50	48	51	*P =* 0·362	48	53	51	58	*P =* 0·525	43	46	45	47	*P =* 0·738	–	*P =* 0·235	*P =* 0·138
Phytoene (µg/d)	2377 (2206)				–	2185 (1916)				–	2561 (2329)				–	***P =*** **0·003**	–	–
	2455	2228	2351	2226	*P =* 0·324	2242	1894	2141	1917	*P =* 0·839	2903	2096	2522	2351	***P =*** **0·032**	–	***P =*** **0·002**	***P =*** **0·086**
Phytofluene (µg/d)	796 (741)				–	739 (631)				–	859 (768)				–	***P =*** **0·001**	–	–
	824	730	789	740	*P =* 0·519	739	672	739	630	*P =* 0·688	954	711	842	782	***P =*** **0·046**	–	***P =*** **0·002**	***P =*** **0·044**
Neoxanthin (µg/d)	458 (354)				–	460 (334)				–	456 (369)				–	*P =* 0·391	–	–
	471	378	453	339	***P =*** **0·046**	465·8	379	455	318	*P =* 0·157	471	361	450	369	*P =* 0·114	–	*P =* 0·463	*P =* 0·395
Violaxanthin (µg/d)	1314 (1172)				–	1242 (1062)				–	1364 (1267)				–	***P =*** **0·008**	–	–
	1277	1206	1326	1172	*P =* 0·952	1256	1175	1241	1032	*P =* 0·541	1414	1283	1364	1275	*P =* 0·801	–	*P =* 0·377	***P =*** **0·009**
Phytoene + phytofluene (µg/d)	3165 (2958)				–	2919 (2535)				–	3428 (3081)				–	***P =*** **0·004**	–	–
	3297	2962	3144	2949	*P =* 0·348	2938	2511	2885	2538	*P =* 0·800	3865	2790	3373	3312	***P =*** **0·034**	–	***P =*** **0·002**	***P =*** **0·092**
Epoxycarotenoids (µg/d)	1779 (1506)				–	1703 (1387)				–	1846 (1590)				–	***P =*** **0·022**	–	–
	1746	1515	1799	1504	*P =* 0·633	1688	1517	1712	1357	*P =* 0·393	1875	15 628	1842	1585	*P =* 0·836	–	*P =* 0·385	***P =*** **0·026**
Total non-provitamin A (µg/d)	11 452 (8162)				–	11 832 (8309)				–	11 252 (7945)				–	*P =* 0·205	–	–
	11 466	8151	11 427	8157	*P =* 0·680	11 440	8285	12 090	8278	*P =* 0·189	11 720	8272	11 172	8084	*P =* 0·558	–	*P =* 0·670	***P =*** **0·063**
Total carotenoids (µg/d)	21 773 (16 785)				–	21 198 (16 538)				–	22 297 (17 418)				–	*P =* 0·635	–	–
	20 820	17 195	22 279	17 303	*P =* 0·237	20 096	13 700	22 072	16 757	***P =*** **0·097**	21 636	17 967	22 519	17 493	*P =* 0·898	–	*P =* 0·230	*P =* 0·716
Fruits (g/d)	295 (274)				–	286 (273)				–	303 (273)				–	*P =* 0·238	–	–
	321	304	281	264	***P =*** **0·003**	310	261	274	257	***P =*** **0·050**	339	302	287	261	***P =*** **0·014**	–	*P =* 0·232	*P =* 0·236
Vegetables (g/d)	218 (172)				–	209 (162)				–	227 (187)				–	***P <*** **0·001**	–	–
	214	171	218	176	*P =* 0·332	199	159	211	161	*P =* 0·605	229	197	226	179	*P =* 0·889	–	***P =*** **0·020**	***P =*** **0·001**
Fruits + vegetables (g/d)	530 (374)				–	495 (365)				–	559 (376)				–	***P =*** **0·003**	–	–
	547	396	526	371	*P =* 0·113	503	370	491	366	*P =* 0·235	606	428	552	361	*P =* 0·108	–	***P =*** **0·049**	***P =*** **0·012**

ORISCAV, Observation des Risques et de la Santé Cardio-Vasculaire au Luxembourg; MetS, metabolic syndrome.

The MetS was diagnosed with the NCEP-ATP III criteria.

MetS [+] and MetS [−] account for the presence and absence of the MetS, respectively.

Total provitamin A corresponds to the sum of *α*-carotene, *β*-carotene and *β*-cryptoxanthin.

Epoxycarotenoids is the sum of neoxanthin and violaxanthin.

Phytoene + phtofluene: the sum of these two carotenoids represents colourless carotenoids.

Total non-provitamin A species corresponds to the sum of lycopene, lutein + zeaxanthin, astaxanthin, phytoene, phytofluene, neoxanthin and violaxanthin.

Total carotenoids correspond to the sum of the total provitamin A carotenoids and total non-provitamin A carotenoids.

Carotenoid intakes were estimated by linking a validated 174-item FFQ to several databases, such as the USDA one.

Independent-samples *t* tests were performed on log-transformed data.

Significant (*P* < 0·05) and tendency (*P* < 0·1) *P*-values are given in bold. *P*-values in blue ink indicate a tendency, that is, *P*-value > 0·05 but < 0·1.

With respect to the consumption of fruits, data showed that participants with MetS ate significantly more fruit items than those without MetS (*P* < 0·01). Such a difference was also observed in men with MetS relative to those without MetS, though results were only marginally significant (*P* = 0·050). However, data showed that vegetables (*P* < 0·001) and fruits + vegetables (*P* < 0·01) were significantly more consumed by women than men (Table 2).

Regarding the intake of carotenoids in persons with *v.* without MetS, no markable differences were observed, even though intakes of neoxanthin and lycopene in persons with MetS were slightly but significantly higher (Table 2).

**Fig. 2.**
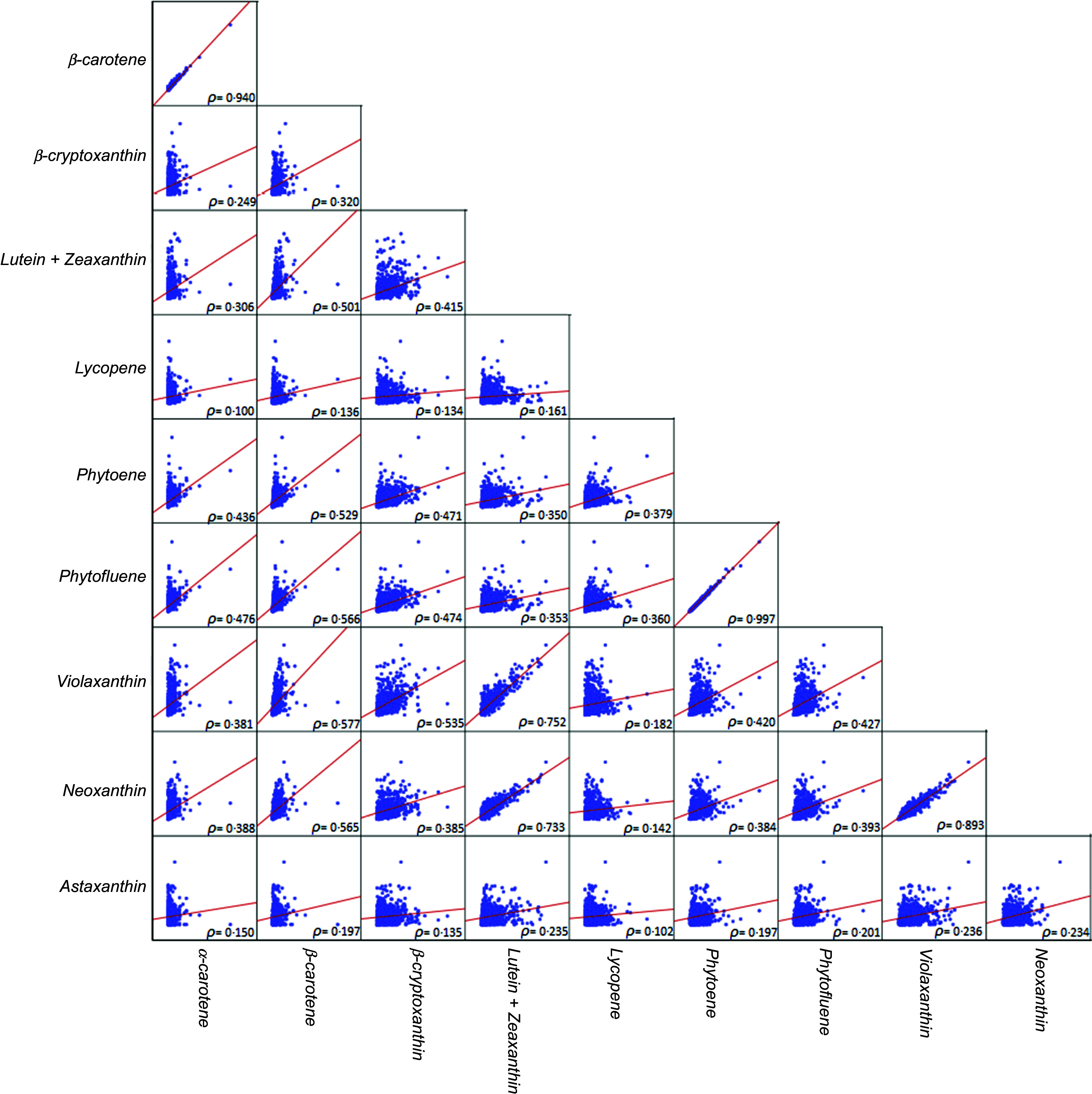
Correlation matrix showing Spearman’s correlation coefficients (ρ) between the intake of individual dietary carotenoids.

As for correlations between individual carotenoids (Fig. 2), the matrix showed that the intakes of all individual carotenoids were significantly correlated (all *P* < 0·001). However, the highest correlations were observed between phytoene and phytofluene (ρ = 0·997) and *α*- and *β*-carotene (ρ = 0·940), and the lowest correlation was between lycopene and *α*-carotene (ρ = 0·100) (Fig. 2).

### Multivariable linear regression analyses

#### Metabolic syndrome and metabolic syndrome scores (fully adjusted)

**Fig. 3.**
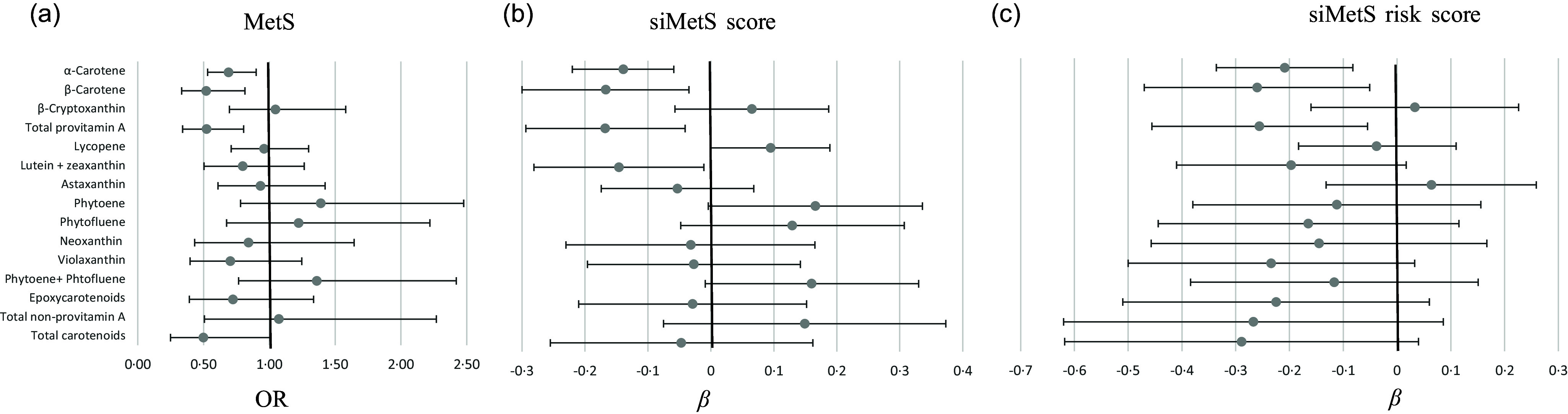
Forest plots showing the association of carotenoid intake on the OR of the metabolic syndrome (MetS) (a) and on the *β* coefficients and its CI of continuous MetS scores, reflecting metabolic status (siMetS score) (b) and metabolic risk (siMetS risk score) (c). The MetS was diagnosed with the NCEP-ATP III criteria. The fully adjusted model (model 4) was employed, adjusting for age, sex, marital status, current smoking status, job status, income, total energy intake, birth country and fruit + vegetable intake. This model was used to study the effect of carotenoids independently from fruit and vegetable intake. Regression analyses for the siMetS risk score did not include sex and age as confounders, as they were included in the formula of this score. Data for logistic regression are expressed as OR with its 95 % CI. Data for linear regression are expressed as *β* regression coefficient with its 95 % CI. NCEP-ATP III, National Cholesterol Education Program – Adult Treatment Panel III.

A marginal significant inverse association was found (*P* = 0·05) after full adjustment for the defined confounders (model 4), for total carotenoid intake and lower odds of MetS (dichotomous outcome, with participants without MetS as the reference group) (Fig. 3(a)). Besides, the fully adjusted binary logistic regression showed significant inverse associations between MetS and the dietary intake of *α*-carotene, *β*-carotene and total provitamin A carotenoids, that is, a protective effect (Fig. 3(a)).

Similarly, the fully adjusted multiple linear regression model indicated significant inverse associations between continuous MetS status (siMetS score) and the intake of these carotenoids (*α*-carotene, *β*-carotene and total provitamin A species) and also that of lutein + zeaxanthin, that is, a protective effect (Fig. 3(b)). The robustness of the fully adjusted regression model was acceptable, with R^2^ ranging from 0·158 to 0·162.

In contrast, a higher lycopene intake was rather predictive of a higher siMetS score (*P* = 0·049, R^2^ = 0·156) (Fig. 3(b)). A tendency towards a positive association between metabolic status (siMetS score) and the intake of phytoene was also found, suggesting that a higher intake of this carotenoid may increase the risk of having severe MetS (Fig. 3(b)). Among all carotenoid species, only a high intake of *α*-carotene, *β*-carotene and total provitamin A carotenoid intakes was significantly associated with lower continuous MetS risk score (Fig. 3(c)). Nevertheless, a tendency towards protective effects against metabolic risk score was also found for violaxanthin, lutein + zeaxanthin and total carotenoid intakes (Fig. 3(c)).

#### Components of metabolic syndrome and additional anthropometric characteristics (fully adjusted)

The fully adjusted multiple linear regression model indicated an inverse association between FBG levels and the intake of total carotenoids, phytoene + phytofluene and *α*-carotene (Table 3). Furthermore, a tendency towards a negative association between FBG levels and either the intake of *β*-carotene, total provitamin A species, phytoene or phytofluene was also found, that is, a protective effect (Table 3).

**Table 3.** Fully adjusted logistic and linear regression models associating carotenoid intake with components of the metabolic syndrome (MetS) and additional anthropometric measurements of Luxembourgish participants (*n* 1346) of the ORISCAV-2 study

Carotenoid intake (µg/d)	Fasting blood glucose (FBG) (mmol/l)	Systolic blood pressure (SBP) (mmHg)	Diastolic blood pressure (DBP) (mmHg)	HDL-cholesterol (mmol/l)	TAG (mmol/l)	Waist circumference (cm)	BMI (kg/m^2^)	WHR
*α*-Carotene	*β* = −0·119 (–0·217, −0·022) ***P*** **= 0·017**	*β* = 0·957 (–0·618, 2·533) *P* = 0·233	*β* = −0·217 (–1·303, 0·869) *P* = 0·695	*β* = 0·044 (0·009, 0·078) ***P*** **= 0·014**	*β* = −0·115 (–0·196, −0·035) ***P*** **= 0·005**	*β* = −1·182 (–2·427, 0·063) ***P*** **= 0·063**	*β* = **−**0·552 (–1·039, −0·064) ***P*** **= 0·027**	*β* = **−**0·008 (–0·015, 0·000) ***P*** **= 0·039**
*β*-Carotene	*β* = −0·157 (–0·318, 0·004) ***P*** **= 0·056**	*β* = 1·108 (–1·494, 3·710) *P* = 0·404	*β* = −0·555 (–2·348, 1·237) *P* = 0·544	*β* = 0·043 (–0·015, 0·100) *P* = 0·146	*β* = −0·146 (–0·279, −0·013) ***P*** **= 0·032**	*β* = −1·274 (–3·332, 0·785) *P* = 0·225	*β* = −0·892 (–1·697, −0·087) ***P*** **= 0·030**	*β* = −0·006 (–0·018, 0·006) *P* = 0·343
*β*-Cryptoxanthin	*β* = 0·024 (–0·124, 0·171) *P* = 0·753	*β* = 0·027 (–2·354, 2·408) *P* = 0·982	*β* = 0·300 (–0·340, 1·941) *P* = 0·720	*β* = −0·002 (–0·054, 0·051) *P* = 0·951	*β* = 0·075 (–0·047, 0·196) *P* = 0·230	*β* = 0·886 (–1·007, 2·778) *P* = 0·359	*β* = 0·446 (–0·292, 1·183) *P* **=**0·236	*β* = 0·001 (–0·010, 0·012) *P* = 0·876
Total provitamin A	*β* = −0·150 (–0·304, 0·004) ***P*** **= 0·056**	*β* = 1·262 (–1·226, 3·751) *P* = 0·320	*β* = −0·452 (–2·168, 1·263) *P* = 0·605	*β* = 0·046 (–0·009, 0·101) *P* = 0·101	*β* = −0·145 (–0·272, −0·018) ***P*** **= 0·026**	*β* = −1·318 (–3·287, 0·651) *P* = 0·189	*β* = **−**0·856 (–1·626, −0·086) ***P*** **= 0·029**	*β* = −0·006 (–0·018, 0·005) *P* = 0·290
Lycopene	*β* = −0·041 (–0·156, 0·074) *P* = 0·486	*β* = −1·112 (–1·960, 1·737) *P* = 0·906	*β* = −0·689 (–1·968, 0·589) *P* = 0·290	*β* = −0·035 (–0·076, 0·006) ***P*** **= 0·099**	*β* = 0·126 (0·032, 0·221) ***P*** **= 0·009**	*β* = 0·190 (–1·276, 1·657) *P* = 0·799	*β* = 0·200 (–0·374, 0·775) *P* **=**0·494	*β* = −0·004 (–0·012, 0·005) *P* = 0·415
Lutein + zeaxanthin	*β* = 0·005 (–0·160, 0·169) *P* = 0·956	*β* = −0·484 (–3·135, 2·167) *P* = 0·720	*β* = −0·482 (–2·308, 1·344) *P* = 0·605	*β* = 0·046 (–0·012, 0·105) *P* = 0·122	*β* = −0·165 (–0·300, −0·029) ***P*** **= 0·017**	*β* = −0·522 (–2·619, 1·575) *P* = 0·626	*β* = −0·281 (–1·103, 0·540) *P* **=**0·502	*β* = −0·004 (–0·016, 0·009) *P* = 0·563
Astaxanthin	*β* = 0·038 (–0·117, 0·192) *P* = 0·630	*β* = −2·317 (–4·776, 0·143) ***P*** **= 0·065**	*β* = −1·305 (–2·997, 0·386) *P* = 0·130	*β* = 0·022 (–0·032, 0·076) *P* = 0·426	*β* = −0·050 (–0·165, 0·065) *P* = 0·396	*β* = 0·038 (–1·919, 1·995) *P* = 0·970	*β* = 0·144 (–0·618, 0·905) *P* = 0·712	*β* = 0·002 (–0·009, 0·014) *P* = 0·697
Phytoene	*β* = −0·173 (–0·379, 0·033) ***P*** **= 0·099**	*β* = −1·929 (–5·260, 1·402) *P* = 0·256	*β* = −2·045 (–4·339, 0·248) ***P*** **= 0·080**	*β* = −0·131 (–0·204, −0·058) ***P < 0·001***	*β* = 0·110 (–0·061, 0·281) *P* = 0·206	*β* = 2·302 (–0·332, 4·936) ***P*** **= 0·087**	*β* = 1·043 (0·011, 2·074) ***P*** **= 0·048**	*β* = 0·016 (0·000, 0·032) ***P*** **= 0·045**
Phytofluene	*β* = −0·205 (–0·420, 0·010) ***P*** **= 0·061**	*β* = −2·842 (–6·311, −0·627) *P* = 0·108	*β* = −2·598 (–4·988, −0·207) ***P*** **= 0·033**	*β* = −0·121 (–0·198, −0·045) ***P*** **= 0·002**	*β* = 0·083 (–0·095, 0·261) *P* = 0·359	*β* = 2·334 (–0·414, 5·083) ***P*** **= 0·096**	*β* = 1·084 (0·008, 2·159) ***P*** **= 0·048**	*β* = 0·016 (–0·001, 0·032) ***P*** **= 0·059**
Neoxanthin	*β* = 0·060 (–0·180, 0·300) *P* = 0·626	*β* = 3·309 (–0·562, 7·179) ***P*** **= 0·094**	*β* = 0·368 (–2·301, 3·038) *P* = 0·787	*β* = 0·000 (–0·086, −0·085) *P* = 0·996	*β* = −0·097 (–0·295, 0·102) *P* = 0·339	*β* = −1·337 (–4·401, 1·726) *P* = 0·392	*β* = −0·727 (–1·927, 0·472) *P* = 0·235	*β* = −0·003 (–0·021, 0·015) *P* = 0·731
Violaxanthin	*β* = 0·037 (–0·168, 0·242) *P* = 0·723	*β* = 1·060 (–2·251, 4·370) *P* = 0·530	*β* = −0·944 (–3·225, 1·336) *P* = 0·417	*β* = −0·017 (–0·091, 0·056) *P* = 0·641	*β* = −0·065 (–0·234, 0·105) *P* = 0·454	*β* = −0·911 (–3·531, 1·708) *P* = 0·495	*β* = −0·775 (–1·800, 0·250) *P* = 0·138	*β* = −0·004 (–0·020, 0·011) *P* = 0·588
Phytoene + phtofluene	*β* = −0·173 (–0·378, −0·033) ***P*** **= 0·009**	*β* = −1·797 (–5·118, 1·525) *P* = 0·289	*β* = −1·997 (–4·284, 0·290) ***P*** **= 0·087**	*β* = −0·127 (–0·200, −0·054) ***P*** **= 0·001**	*β* = 0·106 (–0·064, 0·276) *P* = 0·223	*β* = 2·236 (–0·390, 4·863) ***P*** **= 0·095**	*β* = 1·001 (–0·028, 2·029) ***P*** **= 0·056**	*β* = 0·016 (0·000, 0·031) ***P*** **= 0·048**
Epoxycarotenoids	*β* = 0·049 (–0·171, 0·268) *P* = 0·664	*β* = 1·658 (–1·887, 5·203) *P* = 0·359	*β* = −0·699 (–3·142, 1·744) *P* = 0·575	*β* = −0·015 (–0·093, 0·064) *P* = 0·712	*β* = −0·075 (–0·257, 0·106) *P* = 0·416	*β* = −1·059 (–3·865, 1·746) *P* = 0·459	*β* = **−**0·819 (–1·917, 0·279) *P* = 0·144	*β* = −0·004 (–0·021, 0·012) *P* = 0·605
Total non-provitamin A	*β* = −0·157 (–0·429, 0·115) *P* = 0·259	*β* = −1·565 (–5·960, 2·831) *P* = 0·485	*β* = −2·364 (–5·390, 0·662) *P* = 0·126	*β* = −0·117 (–0·214, −0·020) ***P*** **= 0·018**	*β* = 0·134 (–0·091, 0·359) *P* = 0·242	*β* = 1·278 (–2·199, 4·755) *P* = 0·471	*β* = 0·686 (–0·676, 2·048) *P* = 0·323	*β* = 0·000 (–0·020, 0·021) *P* = 0·966
Total carotenoids	*β* = −0·212 (–0·464, 0·041) ***P*** **= 0·009**	*β* = 0·900 (–3·182, 4·982) *P* = 0·665	*β* = −1·429 (–4·240, 1·383) *P* = 0·319	*β* = −0·008 (–0·099, 0·082) *P* = 0·857	*β* = −0·003 (–0·213, 0·206) *P* = 0·974	*β* = −0·755 (–3·986, 2·476) *P* = 0·647	*β* = **−**0·550 (–1·815, 0·714) *P* = 0·394	*β* = −0·006 (–0·025, 0·013) *P* = 0·546

ORISCAV, Observation des Risques et de la Santé Cardio-Vasculaire au Luxembourg; WHR, waist:hip ratio.

The fully adjusted model (model 4) was used to study the effect of carotenoids independently from fruit and vegetable intake while considering the effects of age, sex, marital status, current smoking status, job status, income, total energy intake, birth country and fruit+vegetable intake.

All predictor variables were log-transformed before multivariable regression models.

Among anthropometric measurements, including BMI and WHR, only waist circumference is considered to be a component of MetS.

Data for logistic regression are expressed as OR with its 95 % CI.

Data for linear regression are expressed as *β* regression coefficient with its 95 % CI.

Total pro-vitamin A species corresponds to the sum of *α*-carotene, *β*-carotene and *β*-cryptoxanthin.

Epoxycarotenoids is the sum of neoxanthin and violaxanthin.

Phytoene+phtofluene: the sum of phytoene and phtofluene represents colourless carotenoids.

Total non-provitamin A species corresponds to the sum of lycopene, lutein+zeaxanthin, astaxanthin, phytoene, phytofluene, neoxanthin and violaxanthin.

Total carotenoids correspond to the sum of the total provitamin A carotenoids and total non-provitamin A carotenoids.

Significant (*P* < 0·05) and tendency (*P* < 0·1) *P*-values are given in bold. *P*-values in blue ink indicate a tendency, that is, *P*-value > 0·05 but < 0·1.

A significant association was found between higher intake of phytofluene and lower DBP, though not for any other carotenoid intake and blood pressure. However, a marginally significant association was found for astaxanthin (inverse association with SBP), neoxanthin (positive association with SBP), phytoene and phytoene + phytofluene (negative associations with DBP), (Table 3).

As for HDL-cholesterol, the fully adjusted multivariable regression model indicated inverse associations between its blood levels and the intakes of lycopene, phytoene, phytofluene, phytoene + phytofluene and total non-provitamin A carotenoids, suggesting potential negative health effects regarding MetS. However, a positive association between HDL-cholesterol levels and carotenoid intake was found only for *α*-carotene, suggesting potential protective health effects regarding MetS.

Regarding TAG, high intakes of *α*-carotene, *β*-carotene, total provitamin A carotenoids and lutein + zeaxanthin were associated with decreased levels of TAG. In contrast, high lycopene intake was positively associated with increased levels of TAG, suggesting potential negative health effects regarding MetS (Table 3).

While high intake of *α*-carotene showed a tendency to protect from central obesity, intakes of phytoene, phytofluene and phytoene + phytofluene had, however, a tendency to be positively associated with waist circumference. As for BMI, not strictly a component of MetS, after full adjustment for confounders, multiple linear regression models indicated significant inverse associations between this anthropometric characteristic and the intake of the *α*-carotene, *β*-carotene, and total provitamin A carotenoids, that is, a protective effect. However, positive associations were found between BMI and intake of phytoene, phytofluene, and tendency for phytoene + phytofluene (Table 3).

A high intake of the same carotenoids, that is, phytoene, in tendency phytofluene and phytoene + phytofluene, was further associated with higher WHR (Table 3). However, high intake of *α*-carotene was associated with lower WHR.

**Fig. 4.**
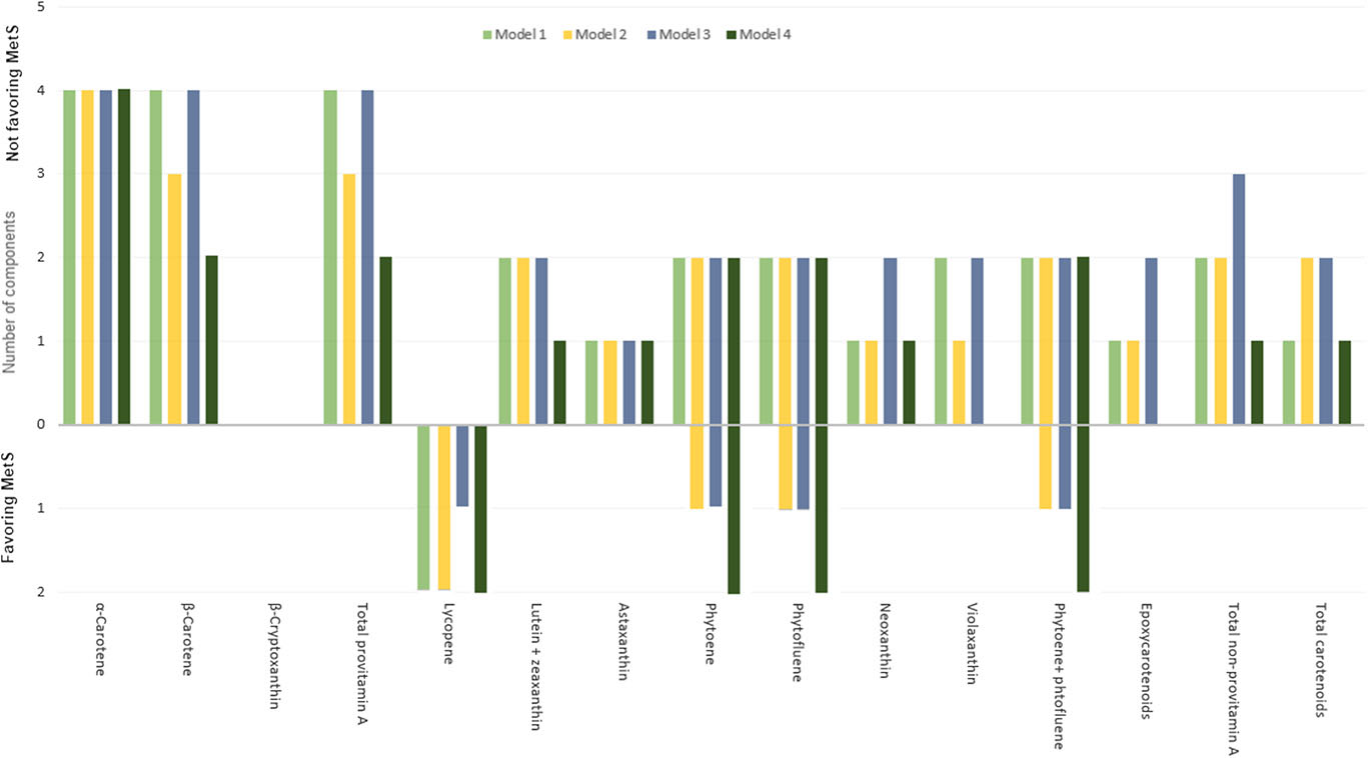
Number of significant associations (and those with a tendency, that is, *P*-value > 0·05 but < 0·1), both inversely related to MetS (bottom part) and those with a positive health impact on MetS (upper part) with the five components of MetS and carotenoid intake, with unadjusted and adjusted linear multivariable regression models. Model 1 was unadjusted; model 2 was adjusted for age and sex; model 3 was further adjusted for marital status, current smoking status, job status, income, total energy intake and birth country; model 4 was further adjusted for fruit + vegetable intake. The five MetS components were central obesity (waist circumference), hypertension (SBP and/or DBP), hyperglycaemia (FBG), TAG and HDL-cholesterol. The number of both associations, either favouring or not MetS, were reported. MetS, metabolic syndrome; SBP, systolic blood pressure; DBP, diastolic blood pressure; FBG, fasting blood glucose.

*α*- and *β*-Carotene and the total sum of provitamin A species were the carotenoids most strongly and inversely associated with a high number of components of MetS, with *α*-carotene being ranked the first, as it was associated with four components in the four models of regression analyses used in this study (Fig. 4). Phytoene, phytofluene and phytoene + phytofluene were also among the carotenoids associated with a high number of MetS components, up to four components in certain regression models (Fig. 4); however, their higher intake was predictive for lower FBG and DBP but also lower HDL-cholesterol and higher waist circumference (Table 3). Lycopene was adversely associated with MetS, significantly associated with two components (Fig. 4), including lower HDL-cholesterol and higher TAG (Table 3).

#### Additional sensitivity analyses – multivariable regression models for lycopene, phytoene and phytofluene containing as confounders tomato-based convenience foods

While analyses showed that higher intake of lycopene predicted severe MetS (siMetS scores), higher TAG and lower HDL-cholesterol (Table 3 for the final adjusted model, and online Supplementary Tables 1–3 for non-adjusted or partially adjusted models), regression analyses considering processed tomato-based food items (pizzas, burgers, ketchup, and lasagna with meat and tomato sauce) as additional confounders demonstrated the absence of any remaining association between lycopene intake and MetS components, in the four models (Table 4 for final adjusted model, and online Supplementary Table 4 for non-adjusted or partially adjusted models). In addition, this adjustment for processed tomato-based items revealed a stronger inverse association of the intake of the colourless carotenoids with FBG and hypertension, particularly in model 3 (Table 4), that is, a protective effect. However, even when adjusting for processed tomato-based items, adverse health associations remained with waist circumference (positive association) and HDL-cholesterol (inverse association), and additional positive associations appeared for BMI and WHR (Table 4).

**Table 4.** Additional sensitivity analysis for fully adjusted models of logistic and linear regression for tomato-based carotenoids (lycopene, phytoene and phytofluene), taking into account potential confounding factors, that is, processed tomato-based food items, associating them with metabolic syndrome (MetS), its scores, components and additional anthropometric measurements of Luxembourgish participants (*n* 1346) of the ORISCAV-2 study

Carotenoid intake (µg/d)	MetS (APT III)	siMetS score (metabolic status)	siMetS risk score (metabolic risk)*	Fasting blood glucose (FBG) (mmol/l)	Systolic blood pressure (SBP) (mmHg)	Diastolic blood pressure (DBP) (mmHg)	HDL-cholesterol (mmol/l)	TAG (mmol/l)	Waist circumference (cm)	BMI (kg/m^2^)	WHR
Lycopene	OR: 0·877 (0·618, 1·244) *P* = 0·462	*β* = 0·091 (–0·023, 0·204) *P* = 0·118	*β* = 0·004 (–0·176, 0·184) *P* = 0·964	*β* = −0·016 (–0·155, 0·122) *P* = 0·817	*β* = −0·170 (–2·408, 2·068) *P* = 0·882	*β* = −0·699 (–2·248, 0·850) *P* = 0·376	*β* = −0·034 (–0·084, 0·015) *P* = 0·173	*β* = 0·101 (–0·013, 0·216) ***P*** **= 0·084**	*β* = 0·635 (–1·134, 2·404) *P* = 0·482	*β* = 0·160 (–0·534, 0·854) *P* = 0·651	*β* = 0·002 (–0·009, 0·012) *P* = 0·715
Phytoene	OR: 1·342 (0·742, 2·426) *P* = 0·330	*β* = 0·135 (–0·038, 0·309) *P* = 0·126	*β* = −0·095 (–0·370, 0·180) *P* = 0·498	*β* = −0·184 (–0·395, 0·026) ***P*** **= 0·086**	*β* = −2·160 (–5·582, 1·262) *P* = 0·216	*β* = −2·066 (–4·425, 0·292) ***P*** **= 0·086**	*β* = −0·126 (–0·201, −0·050) ***P*** **= 0·001**	*β* = 0·086 (–0·107, 0·243) *P* = 0·447	*β* = 2·390 (–0·306, 5·087) ***P*** **= 0·082**	*β* = 0·970 (–0·087, 2·028) ***P*** **= 0·072**	*β* = 0·019 (0·003, 0·035) ***P*** **= 0·018**
Phytofluene	OR: 1·165 (0·631, 2·149) *P* = 0·626	*β* = 0·095 (–0·086, 0·276) *P* = 0·304	*β* = −0·149 (–0·436, 0·137) *P* = 0·307	*β* = −0·222 (–0·442, −0·003) ***P*** **= 0·047**	*β* = −3·191 (–6·753, 0·372) ***P*** **= 0·079**	*β* = −2·669 (–5·126, −0·211) ***P*** **= 0·033**	*β* = −0·115 (–0·194, −0·037) ***P*** **= 0·004**	*β* = 0·039 (–0·143, 0·222) *P* = 0·673	*β* = 2·384 (–0·430, 5·197) ***P*** **= 0·097**	*β* = 1·002 (–0·101, 2·105) ***P*** **= 0·075**	*β* = 0·019 (0·002, 0·036) ***P*** **= 0·026**
Phytoene+phytofluene	OR: 1·311 (0·727, 2·365) *P* = 0·368	*β* = 0·130 (–0·043, 0·303) *P* = 0·141	*β* = −0·100 (–0·374, 0·174) *P* = 0·475	*β* = −0·185 (–0·394, 0·025) ***P*** **= 0·084**	*β* = −2·036 (–5·447, 1·374) *P* = 0·242	*β* = −2·021 (–4·372, 0·329) ***P*** **= 0·092**	*β* = −0·122 (–0·197, −0·047) ***P*** **= 0·001**	*β* = 0·064 (–0·110, 0·239) *P* = 0·471	*β* = 2·309 (–0·378, 4·997) ***P*** **= 0·092**	*β* = 0·925 (–0·129, 1·979) ***P*** **= 0·085**	*β* = 0·019 (0·003, 0·035) ***P*** **= 0·020**

ORISCAV, Observation des Risques et de la Santé Cardio-Vasculaire au Luxembourg; WHR, waist:hip ratio.

* Regression analyses for the siMetS risk score did not include sex and age as confounders, as they were included in the formula of this score.

The MetS was diagnosed with the NCEP-ATP III criteria.

siMetS score and siMetS risk score are continuous MetS scores used to quantify metabolic status and metabolic risk of cardio/cerebrovascular events, respectively.

Model 4: adjusted for age, sex, marital status, current smoking status, job status, income, total energy intake, birth country and fruit + vegetable intake. This model was used to study the effect of carotenoids independently from fruit and vegetable intake.

Sensitivity of the model 4 was increased, considering the following processed tomato-based food items as confounders: pizza, burger, ketchup, and lasagna with meat and tomato sauce.

All predictor variables were log-transformed before multivariable regression models.

Among anthropometric measurements, including BMI and WHR, only waist circumference is considered to be a component of MetS.

Data for logistic regression are expressed as OR with its 95 % CI.

Data for linear regression are expressed as *β* regression coefficient with its 95 % CI.

Total provitamin A species corresponds to the sum of *α*-carotene, *β*-carotene and *β*-cryptoxanthin.

Epoxycarotenoids is the sum of neoxanthin and violaxanthin.

Phytoene + phtofluene: the sum of phytoene and phtofluene represents colourless carotenoids.

Total non-provitamin A species corresponds to the sum of lycopene, lutein + zeaxanthin, astaxanthin, phytoene, phytofluene, neoxanthin and violaxanthin.

Total carotenoids correspond to the sum of the total provitamin A carotenoids and total non-provitamin A carotenoids.

Significant (*P* < 0·05) and tendency (*P* < 0·1) *P*-values are given in bold. *P*-values in blue ink indicate a tendency, that is, *P*-value > 0·05 but < 0·1.

#### Multivariable regression models with fruits and vegetables as independent variables and not confounders

When fruits and vegetables were considered as independent variables (models 1–3, online Supplementary Tables 1–3), associations between the intake of individual carotenoids and total carotenoids, including total provitamin A carotenoids and total non-provitamin A carotenoids, and MetS, its scores, as well as its components, were significantly found. These associations remained significantly associated when fruits and vegetables were considered as confounders (model 4, Table 3).

Adjusted multiple linear regression model without fruits and vegetables as confounders (model 3, fruits and vegetables were considered as independent variables) indicated that fruit consumption was not significantly associated with MetS, its scores, its components and different anthropometric measurements (Table 5). However, high vegetable consumption was inversely associated with lower MetS scores, DBP and BMI, that is, a protective effect. In addition, high vegetable or fruit + vegetable consumption displayed an inverse tendency with FBG, SBP, TAG and waist circumference. Consumption of fruits + vegetables was also negatively associated with metabolic status and metabolic risk (both *P* < 0·05), and in tendency with BMI (Table 5), that is a protective effect.

**Table 5.** Adjusted models of logistic and linear regression associating fruit and vegetable intake with metabolic syndrome (MetS), its scores, components and additional anthropometric measurements of Luxembourgish participants (*n* 1346) of the ORISCAV-2 study

Dietary intakes (g/d)	MetS (APT III)	siMetS score (metabolic status)	siMetS risk score (metabolic risk)*	Fasting blood glucose (FBG) (mmol/l)	Systolic blood pressure (SBP) (mmHg)	Diastolic blood pressure (DBP) (mmHg)	HDL-cholesterol (mmol/l)	TAG (mmol/l)	Waist circumference (cm)	BMI (kg/m^2^)	WHR
Fruits	OR: 1·244 (0·804, 1·925) *P* = 0·326	*β* = −0·076 (–0·201, 0·049) *P* = 0·233	*B* = −0·054 (–0·249, 0·140) *P =* 0·584	*β* = −0·096 (–0·248, 0·055) *P* = 0·212	*β* = −1·054 (–3·496, 1·388) *P* = 0·397	*β* = −0·823 (–2·508, −0·862) *P* = 0·338	*β* = −0·008 (–0·062, −0·046) *P* = 0·763	*β* = −0·083 (–0·209, 0·042) *P* = 0·192	*β* = −0·703 (–2·636, 1·229) *P* = 0·475	*β* = −0·159 (–0·917, 0·599) *P* = 0·682	*β* = −0·004 (–0·016, 0·007) *P* = 0·442
Vegetables	OR: 0·672 (0·402, 1·124) *P* = 0·130	*β* = −0·155 (–0·304, −0·007) ***P*** **= 0·041**	*β* = −0·423 (–0·653, −0·193) ***P <*** **0·001**	*β* = −0·172 (–0·352, 0·009) ***P*** **= 0·063**	*β* = −2·677 (–5·593, 0·238) ***P*** **= 0·072**	*β* = −3·802 (–5·805, −1·798) ***P <*** **0·001**	*β* = −0·006 (–0·070, 0·059) *P* = 0·856	*β* = −0·131 (–0·280, 0·018) ***P*** **= 0·084**	*β* = −2·172 (–4·478, 0·135) ***P*** **= 0·065**	*β* = −1·100 (–2·002, −0·197) ***P*** **= 0·017**	*β* = −0·009 (–0·023, 0·005) *P* = 0·189
Fruits + vegetables	OR: 0·957 (0·525, 1·742) *P* = 0·885	*β* = −0·195 (–0·367, −0·023) ***P*** **= 0·027**	*β* = −0·322 (–0·589, −0·056) ***P =*** **0·018**	*β* = −0·207 (–0·416, 0·002) ***P*** **= 0·053**	*β* = −2·933 (–6·311, 0·445) ***P*** **= 0·089**	*β* = −3·313 (–5·640, −0·986) ***P =*** **0·005**	*β* = 0·002 (–0·073, −0·076) *P* = 0·963	*β* = −0·171 (–0·344, 0·002) ***P*** **= 0·053**	*β* = −2·334 (–5·005, 0·337) ***P*** **= 0·087**	*β* = −0·897 (–1·943, 0·150) ***P*** **= 0·093**	*β* = −0·013 (–0·029, 0·003) *P* = 0·110

ORISCAV, Observation des Risques et de la Santé Cardio-Vasculaire au Luxembourg; WHR: waist:hip ratio.

* Regression analyses for the siMetS risk score did not include sex and age as confounders, as they were included in the formula of this score.

The MetS was diagnosed with the NCEP-ATP III criteria.

siMetS score and siMetS risk score are continuous MetS scores used to quantify metabolic status and metabolic risk of cardio/cerebrovascular events, respectively.

Regression model (model 3) was adjusted for age, sex, marital status, current smoking status, job status, income, total energy intake and birth country.

All predictor variables were log-transformed before multivariable regression models.

Fruits + vegetables correspond to the combined intake of fruits and vegetables.

Among anthropometric measurements, including BMI and WHR, only waist circumference is considered to be a component of MetS.

Data for logistic regression (only for MetS ATP III) are expressed as OR with its 95 % CI.

Data for linear regression are expressed as *β* regression coefficient with its 95 % CI.

Significant (*P* < 0·05) and tendency (*P* < 0·1) *P*-values are given in bold. *P*-values in blue ink indicate a tendency, that is, *P*-value > 0·05 but < 0·1.

## Discussion

The results of our study highlight that carotenoid intake was bidirectionally associated with MetS and its components. A high intake of total carotenoids, in particular of provitamin A species *α*- and *β*-carotene, as well as the non-provitamin A carotenoids lutein + zeaxanthin predicted reduced odds of MetS and its cardiometabolic components, decreased MetS scores (siMetS status score) and metabolic risk (siMetS risk score), as well as anthropometric indicators such as waist circumference, BMI and WHR. By contrast, higher consumptions of the tomato-based carotenoids lycopene, phytoene and phytofluene were rather predictive of lower HDL-cholesterol as well as higher MetS status (siMetS), TAG levels and anthropometric characteristics, possibly due to their relation to a more westernised diet. Moreover, linear regression models showed that these effects persisted, even when considering fruit and vegetable consumption as confounders. To our knowledge, this bidirectional relation has thus far not yet been clearly demonstrated.

Carotenoids are lipophilic pigments occurring in the human diet^(27)^ and may exert several beneficial effects, including improving cardiovascular health^(31)^ and thus reducing the risk of non-communicable diseases^(49–51)^, including MetS^(13,23,52)^. The majority of diet-derived carotenoids originate from vegetables and fruits, even though other sources such as fish, eggs and milk may contribute to a lesser extent to their dietary intakes^(31,44,45)^. Researchers have reported significant positive associations between their intake and blood concentrations, and *β*-carotene and lycopene are among the most frequently consumed dietary carotenoids and display the highest circulating concentrations of carotenoids^(20,53)^.

The present findings showed that *β*-carotene was the most frequently consumed carotenoid (6·5 mg/d), followed by lycopene (3·6 mg/d) and *α*-carotene (2·5 mg/d) (Table 2). A high correlation was found between *α*-and *β*-carotene (ρ = 0·94), indicating that *β*-carotene-rich foods are also a good source of *α*-carotene (Fig. 2). However, the correlation between lycopene and *α*-and *β*-carotene was rather weak (ρ = 0·10 and ρ = 0·14, respectively), indicating different food sources of these carotenoids. Compared with men, women’s median daily intakes of *β*-carotene (6·9 *v*. 6·1 mg) and *α*-carotene (2·6 *v*. 2·4 mg) were significantly higher, while lycopene consumption was significantly lower (3·2 *v*. 4·2 mg) (Table 2). Consistent with the literature, it has been reported that women consume higher amounts of provitamin A species than men, resulting in higher blood concentrations^(20)^, and this correlation could likely be stronger when considering the higher daily energy intake in men than in women^(54)^. Regarding lycopene, both higher and lower concentrations in blood have been reported in men *v*. women^(20)^.

Carotenoids may reflect a diet rich in fruits and vegetables^(31–33)^, which would at least contribute to the observed health benefits, following additive or synergist actions of complex mixtures of non-nutrients and nutrients^(27,34)^. However, independently from fruit and vegetable intake, the fully adjusted logistic regression model indicated that total carotenoids and total and individual provitamin A species decreased the odds of MetS. A higher dietary intake of these carotenoids, including *α*-and *β*-carotene, reduced 31 % to 48 % of the odds of the MetS, respectively (Fig. 3(a)). This is in agreement with the results of Beydoun et al.^(13)^, indicating an inverse correlation between MetS and serum total carotenoids, with the strongest one observed for *β*-carotene, followed by *α*-carotene. In another recent meta-analysis of observational studies, MetS with disturbances in lipid metabolism was linked to reduced serum levels of carotenoids, with the strongest reduction for *β*-carotene^(23)^. However, the lowest relation was found for *α*-carotene and *β*-cryptoxanthin^(23)^. In a cross-sectional study, it has been reported that low levels of serum carotenoids were associated with higher odds of MetS as well as with an increasing number of any of the five MetS components being present^(55)^. In this cross-sectional study, *α*- and *β*-carotene and the total sum of provitamin A species were the carotenoids most strongly and inversely associated with the number of components of MetS (Fig. 4). In light of the above, it can be suggested that carotenoids individually (e.g. *β*-carotene) and collectively (e.g. total provitamin A carotenoids) appeared to be protective against the incidence of MetS.

However, most often, the diagnosis of MetS is based on a dichotomous decision (presence or not), which may result in the loss of valuable information on the metabolic status of participants, such as those with borderline values or with < 3 positive cardiometabolic risk factors, conditions that impede the clinical diagnosis of MetS. Thus, a validated continuous score of MetS (siMetS score) was calculated to quantify metabolic status, as well as the degree and severity of cardiometabolic affliction in all participants, including those not diagnosed with MetS^(46,47)^. Findings indicated that the intake of total and individual provitamin A carotenoids (*α*- and *β*-carotene) were inversely related to metabolic status (siMetS score) (Fig. 3(b)). In accordance with these findings, daily high consumption of these carotenoids were related to a higher protection of participants from developing high BMI and higher levels of cardiometabolic components of MetS, including FBG and TAG (Table 3). Furthermore, the metabolic risk (siMetS risk score) was quantified for participants, considering both age and heredity (family background of cardiovascular events). The findings indicated that daily intake of total and individual provitamin A carotenoids (*α*- and *β*-carotene) significantly reduced the severity of MetS (Fig. 3(b)) and also its risk (Fig. 3(c)), while a low intake enhanced severity and risk.

MetS (dichotomous outcome) was not significantly related to *β*-cryptoxanthin or any non-provitamin A carotenoids, as well as total carotenoids, despite an existing tendency for the latter. However, as for continuous dependent variables, marginal, even significant beneficial effects of the combined intakes of lutein and zeaxanthin on MetS status score (siMetS), siMetS risk score and TAG were found. Lutein and zeaxanthin can be considered as indicators of a healthier diet, as the major sources of these carotenoids are leafy green vegetables such as broccoli, peas and green beans^(44)^. Several health benefits have been attributed to lutein and zeaxanthin, including effects on eye health and cardiometabolic health^(23,52,56)^. For example, a meta-analysis of observational studies^(23)^ showed that low blood concentrations of lutein + zeaxanthin were associated with developing MetS, concomitantly with disturbances in lipid metabolism. Another meta-analysis of trials, cohort, cased control and cross-sectional studies showed that higher lutein intake or blood concentrations were related to a reduced likelihood of developing stroke, CHD and MetS^(52)^. Interestingly, there is some evidence that the combined dietary intake of lutein/zeaxanthin, circulating concentrations of lutein/zeaxanthin or lutein alone were related to high levels of physical activity and inversely with sedentary behaviour^(57–60)^. The relationship with physical activity was thought to be causative, which would warrant further investigation of the action of lutein-rich vegetables, possibly related to positive changes in lifestyle factors, following its direct action on the brain, though this remains speculative^(57–59)^.

Astaxanthin is a red pigment mostly consumed via aquatic species such as salmon, trout, shrimp and crustaceans^(45,61)^. In the ORISCAV-LUX-2 study, astaxanthin was the lowest-consumed carotenoid by participants (median of 47 µg/d). Several health benefits have been attributed to this carotenoid, including its protective role on the cardiovascular system^(61,62)^. Leung et al.^(61)^ pointed out in a meta-analysis of randomised controlled trials its protective effect on individuals at risk of MetS, reducing in tendency total cholesterol, SBP, and at a significant level, LDL-cholesterol. The fully adjusted linear regression model also indicated a marginal significant and, thus, protective effect of astaxanthin on SBP (*P* = 0·06) (Table 3). However, when fruits and vegetables were not considered as confounders, a significant inverse association between astaxanthin intake and SBP, as well as marginal on DBP, was found (online Supplementary Table 3). Due to the very low intake of astaxanthin, likely, this component acted primarily as an indicator for the intake of certain types of fish and seafood, such as trout and shrimp, which have been inversely associated with MetS^(63–65)^.

In this study, women consumed significantly more fruits, vegetables and fruits + vegetables than men. Linear regression models showed that either vegetable or fruit + vegetable consumption were inversely associated with MetS scores and its components, that is, suggesting a protective effect (Table 5). However, fruits alone failed to demonstrate an inverse relation with MetS and its components in the adjusted models. The unadjusted model even indicated a positive relation to MetS in the participants (online Supplementary Table 1), which could be related to the high sugar content in all fruit items or be due to inverse causality, that is, that persons with MetS made sure to consume sufficient fruits per d.

Most importantly, findings from the present study suggested that lycopene, phytoene and phytofluene were positively associated with MetS severity (continuous scale) (Fig. 3(b)). These carotenoids have been especially associated with convenient, processed and fast food containing processed tomato products such as pizza, ketchup, sauces and burgers. Thus, unlike *α*- and *β*-carotene, lutein and zeaxanthin, lycopene, and to a lesser degree, due to lack of data, phytoene and phytofluene, have been associated with rather westernised dietary patterns^(20)^. For example, 5 g of ketchup may contain 439 µg of lycopene, which is at least three times higher than that of raw tomato^(19)^, not yet considering also its much higher bioavailability^(66)^. The fully adjusted linear regression model showed that higher intake of lycopene was predictive of higher metabolic severity (siMetS) score and TAG, as well as lower levels of HDL-cholesterol. It is worth noting that the consumption of lycopene-rich tomatoes in some studies was related to reduced likelihood of several chronic diseases, including CVD, for which the risk may diminish due to, for example, cholesterol-lowering effects^(35,67)^. Likewise, elevated serum lycopene levels or intake were associated with a reduced risk of MetS^(23,37)^. In addition, a meta-analysis of human interventional trials revealed that lycopene intake significantly ameliorated HDL-cholesterol levels^(36)^. Therefore, adverse effects of lycopene on the metabolic health of participants of the ORISCAV-LUX-2 study may be attributed to its indicator properties for high consumption of processed tomato-based foods and thus as a marker of a rather westernised diet. However, when pizzas, burgers, ketchup, and lasagna with meat and tomato sauce were included as confounders in the model, no physiologically adverse associations remained between lycopene consumption and the severity of MetS and its components (Table 4). This suggests that lycopene by itself is not a ‘bad’ carotenoid, but it is rather a valuable indicator for unhealthy/westernised diets. In general, it has been pointed out that dietary patterns in Luxembourg, as in other westernised countries, have been developing towards more westernised patterns, marked by lower fruit and vegetable intake and increased intake of meat, sauces, drinks, and fast, processed, and convenient foods^(42)^ rich in salt, sugar, fat, and additives, and characterised by low quantities of micronutrients^(3,68)^. These aspects may have suppressed any beneficial direct effects that lycopene may have had on the subjects in this study.

Phytoene and phytofluene are typically not included in food carotenoid databases despite their large presence in plant-based foods such as oranges, prunes, apricots, peaches, watermelon, and especially tomatoes and tomato products^(35,69)^. These precursors of other carotenoids are found in human samples, including plasma, milk and tissues^(69)^. However, epidemiological evidence is missing to confirm their putative health advantages observed in *in vitro* and animal studies^(69)^. In the present study, the median daily intake of the colourless phytoene and phytofluene in participants (3·2 mg) was only slightly lower than that of lycopene (3·6 mg) (Table 2). Even though the fully adjusted logistic regression models did not confirm any significant association with MetS, higher intake of phytoene and/or phytofluene was positively associated with metabolic severity score and anthropometric measures, including waist circumference, BMI and WHR. In addition, significant inverse associations were found between phytoene and/or phytofluene consumption and HDL-cholesterol. These results suggest that phytoene and phytofluene may be, similarly as to lycopene, associated with westernised dietary patterns, as they also occur in lycopene-rich foods such as ketchup/pizza. However, consumption of these colourless carotenoids was found to be inversely associated with FBG and DBP, that is, a protective effect. The fact that these colourless carotenoids still presented adverse associations with MetS components even after adjusting for processed tomato products containing foods (Table 4) may suggest a differential action of phytoene/phytofluene or merely reflect a broader dietary origin compared with lycopene. This is in line with the modest correlation found between lycopene and both phytoene (ρ = 0·38) and phytofluene (ρ = 0·36) (Fig. 2), suggesting that lycopene-rich foods are only a part of food items that supplied these colourless carotenoids for participants of ORISCAV-LUX-2 survey.

The main strength of this study is the combination of various databases for completing carotenoid containing food items, with the most frequently consumed carotenoids being included. Several potential confounders were considered, including fruit and vegetable intake. The number of participants in this study is fairly high, especially given that Luxembourg is a small country. In addition, the FFQ was applied by trained nurses. However, the main limitation of this investigation is capturing food intakes by FFQ, as this technique – as other dietary recalls, is prone to recall bias^(70)^. In addition, the cross-sectional nature of this study does not allow for drawing causative conclusions. Another limitation of our study is the lack of data regarding the study participants’ use of carotenoid supplements, as well as the lack of serum measurements related to the intake of carotenoids. It is essential to consider these factors in future studies to investigate the effects of carotenoids comprehensively.

In conclusion, this study showed divergent effects of carotenoid intake on metabolic status, risk and syndrome, and its cardiometabolic components in ORISCAV-LUX-2 participants, depending on the carotenoid type. While *α*- and *β*-carotene, total provitamin A carotenoids, and lutein + zeaxanthin were associated with improved MetS-related health outcomes, high intake of lycopene, phytoene and phytofluene were associated with worse outcomes. As lycopene and uncoloured carotenoids dietary sources in a westernised diet (same as our study) are mainly from processed tomato-containing foods, including pizza, burgers, puree, sauces and ketchup, they may be considered as new indicators of an unhealthy and westernised dietary pattern. Future investigations are warranted to explore the role of lycopene and the uncoloured carotenoids phytoene and phytofluene as markers of dietary patterns or their direct impact on components of the MetS.

## Supporting information

Bouayed and Vahid supplementary material Bouayed and Vahid supplementary material

## Supplementary material

For supplementary material/s referred to in this article, please visit https://doi.org/10.1017/S0007114524000758

## References

[1] Dominguez LJ , Di Bella G , Veronese N , et al. (2021) Impact of Mediterranean diet on chronic non-communicable diseases and longevity. Nutrients 13, 2028. 34204683 10.3390/nu13062028 PMC8231595

[2] Dreher ML (2018) Whole fruits and fruit fiber emerging health effects. Nutrients 10, 1833. 30487459 10.3390/nu10121833 PMC6315720

[3] Bouayed J & Bohn T (2022) Nine ‘brain food’ tips for researchers. Nature. doi: 10.1038/d41586-022-00763-7. 35296837

[4] Elizabeth L , Machado P , Zinöcker M , et al. (2020) Ultra-processed foods and health outcomes: a narrative review. Nutrients 12, 1955. 32630022 10.3390/nu12071955 PMC7399967

[5] Bohn T , Samouda H & Alkerwi AA (2022) Chapter 7 - Dietary patterns and type 2 diabetes—relationship to metabolic syndrome and inflammation. In Diet, Inflammation, and Health, pp. 261–366 [ JR Hébert and LJ Hofseth , editors]. Academic Press. doi: 10.1016/B978-0-12-822130-3.00014-4.

[6] Balkau B , Valensi P , Eschwège E , et al. (2007) A review of the metabolic syndrome. Diabetes Metab 33, 405–413. 17981485 10.1016/j.diabet.2007.08.001

[7] Saklayen MG (2018) The global epidemic of the metabolic syndrome. Curr Hypertens Rep 20, 12. 29480368 10.1007/s11906-018-0812-z PMC5866840

[8] Zuin M , Roncon L , Passaro A , et al. (2021) Metabolic syndrome and the risk of late onset Alzheimer’s disease: an updated review and meta-analysis. Nutr Metab Cardiovasc Dis 31, 2244–2252. 34039508 10.1016/j.numecd.2021.03.020

[9] Lifshitz K , Ber Y & Margel D (2021) Role of metabolic syndrome in prostate cancer development. Eur Urol Focus 7, 508–512. 33994167 10.1016/j.euf.2021.04.022

[10] Teklu M , Zhou W , Kapoor P , et al. (2021) Metabolic syndrome and its factors are associated with noncalcified coronary burden in psoriasis: an observational cohort study. J Am Acad Dermatol 84, 1329–1338. 33383084 10.1016/j.jaad.2020.12.044

[11] Alkerwi A , Donneau AF , Sauvageot N , et al. (2011) Prevalence of the metabolic syndrome in Luxembourg according to the Joint Interim Statement definition estimated from the ORISCAV-LUX study. BMC Public Health 11, 4. 21205296 10.1186/1471-2458-11-4 PMC3024931

[12] Eckel RH , Grundy SM & Zimmet PZ (2005) The metabolic syndrome. Lancet 365, 1415–1428. 15836891 10.1016/S0140-6736(05)66378-7

[13] Beydoun MA , Chen X , Jha K , et al. (2019) Carotenoids, vitamin A, and their association with the metabolic syndrome: a systematic review and meta-analysis. Nutr Rev 77, 32–45. 30202882 10.1093/nutrit/nuy044 PMC6277204

[14] Gesteiro E , Megía A , Guadalupe-Grau A , et al. (2021) Early identification of metabolic syndrome risk: a review of reviews and proposal for defining pre-metabolic syndrome status. Nutr Metab Cardiovasc Dis 31, 2557–2574. 34244048 10.1016/j.numecd.2021.05.022

[15] van’t Erve TJ , Kadiiska MB , London SJ , et al. (2017) Classifying oxidative stress by F(2)-isoprostane levels across human diseases: a meta-analysis. Redox Biol 12, 582–599. 10.1016/j.redox.2017.03.024 PMC5384299 28391180

[16] Zhang Y & Zhang DZ (2018) Associations of vegetable and fruit consumption with metabolic syndrome. A meta-analysis of observational studies. Public Health Nutr 21, 1693–1703. 29506604 10.1017/S1368980018000381 PMC10261586

[17] Tian Y , Su L , Wang J , et al. (2018) Fruit and vegetable consumption and risk of the metabolic syndrome: a meta-analysis. Public Health Nutr 21, 756–765. 29151369 10.1017/S136898001700310X PMC10260986

[18] Bohn T (2019) Carotenoids and markers of oxidative stress in human observational studies and intervention trials: implications for chronic diseases. Antioxidants (Basel) 8, 179. 31213029 10.3390/antiox8060179 PMC6616644

[19] Biehler E , Alkerwi AA , Hoffmann L , et al. (2012) Contribution of violaxanthin, neoxanthin, phytoene and phytofluene to total carotenoid intake: assessment in Luxembourg. J Food Compos Anal 25, 56–65.

[20] Böhm V , Lietz G , Olmedilla-Alonso B , et al. (2021) From carotenoid intake to carotenoid blood and tissue concentrations – implications for dietary intake recommendations. Nutr Rev 79, 544–573. 32766681 10.1093/nutrit/nuaa008 PMC8025354

[21] McKay GJ , Lyner N , Linden GJ , et al. (2021) Association of low plasma antioxidant levels with all-cause mortality and coronary events in healthy middle-aged men from France and Northern Ireland in the PRIME study. Eur J Nutr 60, 2631–2641. 33355688 10.1007/s00394-020-02455-2 PMC8275518

[22] Białkowska A , Górnicka M , Zielinska-Pukos MA , et al. (2023) Plasma carotenoids and polyphenols and their association with MetS: the need for nutritional interventions. Antioxidants 12, 1336. 10.3390/antiox12071336 PMC10376012 37507876

[23] Iqbal WA , Mendes I , Finney K , et al. (2021) Reduced plasma carotenoids in individuals suffering from metabolic diseases with disturbances in lipid metabolism: a systematic review and meta-analysis of observational studies. Int J Food Sci Nutr 72, 879–891. 33586569 10.1080/09637486.2021.1882962

[24] Bonet ML , Canas JA , Ribot J , et al. (2015) Carotenoids and their conversion products in the control of adipocyte function, adiposity and obesity. Arch Biochem Biophys 572, 112–125. 25721497 10.1016/j.abb.2015.02.022

[25] Bonet ML , Canas JA , Ribot J , et al. (2016) Carotenoids in adipose tissue biology and obesity. Subcell Biochem 79, 377–414. 27485231 10.1007/978-3-319-39126-7_15

[26] Bohn T , Bonet ML , Borel P , et al. (2021) Mechanistic aspects of carotenoid health benefits – where are we now? Nutr Res Rev 34, 276–302. 34057057 10.1017/S0954422421000147

[27] Bouayed J & Bohn T (2012) Chapter 1 – Dietary derived antioxidants: implications on health. In Nutrition, Well-Being and Health, pp. 1–22 [ J Bouayed and T Bohn , editors]. Rijeka, Croatia: Intech.

[28] Eroglu A , Al’Abri IS , Kopec RE , et al. (2023) Carotenoids and their health benefits as derived via their interactions with gut microbiota. Adv Nutr 14, 238–255. 36775788 10.1016/j.advnut.2022.10.007 PMC10229386

[29] Bohn T , de Lera AR , Landrier JF , et al. (2022) Carotenoid metabolites, their tissue and blood concentrations in humans and further bioactivity via retinoid receptor-mediated signalling. Nutr Res Rev 36, 498–511. 36380523 10.1017/S095442242200021X

[30] Zheng JS , Sharp SJ , Imamura F , et al. (2020) Association of plasma biomarkers of fruit and vegetable intake with incident type 2 diabetes: EPIC-InterAct case-cohort study in eight European countries. BMJ 370, m2194. 32641421 10.1136/bmj.m2194 PMC7341350

[31] Voutilainen S , Nurmi T , Mursu J , et al. (2006) Carotenoids and cardiovascular health. Am J Clin Nutr 83, 1265–1271. 16762935 10.1093/ajcn/83.6.1265

[32] Bationo JF , Zeba AN , Abbeddou S , et al. (2018) Serum carotenoids reveal poor fruit and vegetable intake among schoolchildren in Burkina Faso. Nutrients 10, 1422. 30287727 10.3390/nu10101422 PMC6213241

[33] Ziegler RG (1991) Vegetables, fruits, and carotenoids and the risk of cancer. Am J Clin Nutr 53, 251s–259s. 1985395 10.1093/ajcn/53.1.251S

[34] Bouayed J & Bohn T (2010) Exogenous antioxidants--double-edged swords in cellular redox state: health beneficial effects at physiologic doses *v.* deleterious effects at high doses. Oxid Med Cell Longev 3, 228–237. 20972369 10.4161/oxim.3.4.12858 PMC2952083

[35] Engelmann NJ , Clinton SK & Erdman JW Jr (2011) Nutritional aspects of phytoene and phytofluene, carotenoid precursors to lycopene. Adv Nutr 2, 51–61. 22211189 10.3945/an.110.000075 PMC3042793

[36] Inoue T , Yoshida K , Sasaki E , et al. (2021) Effects of lycopene intake on HDL-cholesterol and triglyceride levels: a systematic review with meta-analysis. J Food Sci 86, 3285–3302. 34268742 10.1111/1750-3841.15833

[37] Sluijs I , Beulens JWJ , Grobbee DE , et al. (2009) Dietary carotenoid intake is associated with lower prevalence of metabolic syndrome in middle-aged and elderly men. J Nutr 139, 987–992. 19321578 10.3945/jn.108.101451

[38] Fabiani R , Naldini G & Chiavarini M (2019) Dietary patterns and metabolic syndrome in adult subjects: a systematic review and meta-analysis. Nutrients 11, 2056. 31480732 10.3390/nu11092056 PMC6770202

[39] Rodríguez-Monforte M , Sánchez E , Barrio F , et al. (2017) Metabolic syndrome and dietary patterns: a systematic review and meta-analysis of observational studies. Eur J Nutr 56, 925–947. 27605002 10.1007/s00394-016-1305-y

[40] Alkerwi A , Pastore J , Sauvageot N , et al. (2019) Challenges and benefits of integrating diverse sampling strategies in the observation of cardiovascular risk factors (ORISCAV-LUX 2) study. BMC Med Res Methodol 19, 27. 30717671 10.1186/s12874-019-0669-0 PMC6360765

[41] Alkerwi A , Sauvageot N , Donneau AF , et al. (2010) First nationwide survey on cardiovascular risk factors in Grand-Duchy of Luxembourg (ORISCAV-LUX). BMC Public Health 10, 468. 20698957 10.1186/1471-2458-10-468 PMC2925827

[42] Vahid F , Brito A , Le Coroller G , et al. (2021) Dietary intake of adult residents in Luxembourg taking part in two cross-sectional studies-ORISCAV-LUX (2007–2008) and ORISCAV-LUX 2 (2016–2017). Nutrients 13, 4382. 10.3390/nu13124382 PMC8706514 34959934

[43] Vahid F , Hoge A , Hébert JR , et al. (2023) Association of diet quality indices with serum and metabolic biomarkers in participants of the ORISCAV-LUX-2 study. Eur J Nutr 62, 2063–2085. 36917281 10.1007/s00394-023-03095-y PMC10349755

[44] Holden JM , Eldridge AL , Beecher GR , et al. (1999) Carotenoid content of U.S. foods: an update of the database. J Food Compos Anal 12, 169–196.

[45] EFSA (2005) Opinion of the scientific panel on additives and products or substances used in animal feed on the request from the European commission on the safety of use of colouring agents in animal nutrition PART I. General principles and astaxanthin. EFSA J 291, 1–40.

[46] Sebekova K & Sebek J (2018) Continuous metabolic syndrome score (siMS) enables quantification of severity of cardiometabolic affliction in individuals not presenting with metabolic syndrome. Bratisl Lek Listy 119, 675–678. 30672711 10.4149/BLL_2018_121

[47] Soldatovic I , Vukovic R , Culafic D , et al. (2016) siMS score: simple method for quantifying metabolic syndrome. PLoS One 11, e0146143. 26745635 10.1371/journal.pone.0146143 PMC4706421

[48] Al Kudsee K , Vahid F & Bohn T (2022) High adherence to the Mediterranean diet and Alternative Healthy Eating Index are associated with reduced odds of metabolic syndrome and its components in participants of the ORISCAV-LUX2 study. Front Nutr 9, 1087985. 36583217 10.3389/fnut.2022.1087985 PMC9793091

[49] Beydoun MA , Beydoun HA , Fanelli-Kuczmarski MT , et al. (2022) Association of serum antioxidant vitamins and carotenoids with incident Alzheimer disease and all-cause dementia among US adults. Neurology 98, e2150–e2162. 35508396 10.1212/WNL.0000000000200289 PMC9169941

[50] Jiang YW , Sun ZH , Tong WW , et al. (2021) Dietary intake and circulating concentrations of carotenoids and risk of type 2 diabetes: a dose-response meta-analysis of prospective observational studies. Adv Nutr 12, 1723–1733. 33979433 10.1093/advances/nmab048 PMC8483954

[51] Cheng HM , Koutsidis G , Lodge JK , et al. (2019) Lycopene and tomato and risk of cardiovascular diseases: a systematic review and meta-analysis of epidemiological evidence. Crit Rev Food Sci Nutr 59, 141–158. 28799780 10.1080/10408398.2017.1362630

[52] Leermakers ET , Darweesh SK , Baena CP , et al. (2016) The effects of lutein on cardiometabolic health across the life course: a systematic review and meta-analysis. Am J Clin Nutr 103, 481–494. 26762372 10.3945/ajcn.115.120931

[53] Bohn T , Desmarchelier C , El SN , et al. (2019) *β*-Carotene in the human body: metabolic bioactivation pathways - from digestion to tissue distribution and excretion. Proc Nutr Soc 78, 68–87. 30747092 10.1017/S0029665118002641

[54] Bennett E , Peters SAE & Woodward M (2018) Sex differences in macronutrient intake and adherence to dietary recommendations: findings from the UK Biobank. BMJ Open 8, e020017. 10.1136/bmjopen-2017-020017 PMC5922487 29691247

[55] Kanagasabai T , Alkhalaqi K , Churilla JR , et al. (2019) The association between metabolic syndrome and serum concentrations of micronutrients, inflammation, and oxidative stress outside of the clinical reference ranges: a cross-sectional study. Metab Syndr Relat Disord 17, 29–36. 30372368 10.1089/met.2018.0080

[56] Mares J (2016) Lutein and zeaxanthin isomers in eye health and disease. Annu Rev Nutr 36, 571–602. 27431371 10.1146/annurev-nutr-071715-051110 PMC5611842

[57] Crichton G , Elias M , Alkerwi A , et al. (2015) Intake of lutein-rich vegetables is associated with higher levels of physical activity. Nutrients 7, 8058–8071. 26393650 10.3390/nu7095378 PMC4586573

[58] Rock CL , Thornquist MD , Neuhouser ML , et al. (2002) Diet and lifestyle correlates of lutein in the blood and diet. J Nutr 132, 525s–530s. 11880586 10.1093/jn/132.3.525S

[59] Gruber M , Chappell R , Millen A , et al. (2004) Correlates of serum lutein + zeaxanthin: findings from the Third National Health and Nutrition Examination Survey. J Nutr 134, 2387–2394. 15333733 10.1093/jn/134.9.2387

[60] Thomson RL , Coates AM , Howe PR , et al. (2014) Increases in plasma lutein through supplementation are correlated with increases in physical activity and reductions in sedentary time in older adults. Nutrients 6, 974–984. 24594505 10.3390/nu6030974 PMC3967172

[61] Leung LY , Chan SM , Tam HL , et al. (2022) Astaxanthin influence on health outcomes of adults at risk of metabolic syndrome: a systematic review and meta-analysis. Nutrients 14, 2050. 35631193 10.3390/nu14102050 PMC9148008

[62] Donoso A , González-Durán J , Muñoz AA , et al. (2021) Therapeutic uses of natural astaxanthin: an evidence-based review focused on human clinical trials. Pharmacol Res 166, 105479. 33549728 10.1016/j.phrs.2021.105479

[63] Tørris C , Småstuen MC & Molin M (2018) Nutrients in fish and possible associations with cardiovascular disease risk factors in metabolic syndrome. Nutrients 10, 952. 30041496 10.3390/nu10070952 PMC6073188

[64] Tørris C , Molin M & Cvancarova Småstuen M (2014) Fish consumption and its possible preventive role on the development and prevalence of metabolic syndrome – a systematic review. Diabetol Metab Syndr 6, 112. 25352919 10.1186/1758-5996-6-112 PMC4210541

[65] Karimi G , Heidari Z , Firouzi S , et al. (2020) A systematic review and meta-analysis of the association between fish consumption and risk of metabolic syndrome. Nutr Metab Cardiovasc Dis 30, 717–729. 32127332 10.1016/j.numecd.2020.02.001

[66] Unlu NZ , Bohn T , Francis DM , et al. (2007) Lycopene from heat-induced cis-isomer-rich tomato sauce is more bioavailable than from all-trans-rich tomato sauce in human subjects. Br J Nutr 98, 140–146. 17391568 10.1017/S0007114507685201

[67] Løchen M-L (2023) One tomato a day may keep the doctor away. Eur J Prev Cardiol. zwad393. doi: 10.1093/eurjpc/zwad393. 38113394

[68] Ifland JR , Preuss HG , Marcus MT , et al. (2009) Refined food addiction: a classic substance use disorder. Med Hypotheses 72, 518–526. 19223127 10.1016/j.mehy.2008.11.035

[69] Meléndez-Martínez AJ , Mapelli-Brahm P , Benítez-González A , et al. (2015) A comprehensive review on the colorless carotenoids phytoene and phytofluene. Arch Biochem Biophys 572, 188–200. 25615528 10.1016/j.abb.2015.01.003

[70] Food and Agriculture Organization (FAO) (2018) *Dietary Assessment: A Resource Guide to Method Selection and Application in Low Resource Settings*. pp 1–172. Rome, Italy: Food and Agriculture Organization (FAO).

