# Carotenoid pattern intake and relation to metabolic status, risk and syndrome, and its components – divergent findings from the ORISCAV-LUX-2 survey – ERRATUM

**DOI:** 10.1017/S0007114524001016

**Published:** 2024-07-14

**Authors:** Jaouad Bouayed, Farhad Vahid

In the original publication α- and β- for carotene species was missing in the graphical abstract. This has been amended in the original article.

The publisher apologises for this error.

