# Supplementary material for: Carotenoid pattern intake and relation to metabolic status, risk and syndrome, and its components – divergent findings from the ORISCAV-LUX-2 survey
Source: Br J Nutr. 2024 Apr 19;132(1):50–66. doi: 10.1017/S0007114524000758 (PMC11420883; doi:10.1017/S0007114524000758)
Supplement: Bouayed and Vahid supplementary material [file S0007114524000758sup001.docx]

**Supplementary table 1.** Unadjusted logistic and linear regression models associating carotenoid, fruit and vegetable intake with metabolic syndrome (MetS), its scores, components and additional anthropometric measurements of Luxembourgish participants (n=1346) of the ORISCAV-2 study.

| **Carotenoid intake**  **(µg /day)** | **MetS (APT III)** | **siMetS score (metabolic status)** | **siMetS risk score (metabolic risk)** | **Fasting blood glucose (FBG) (mmol /L)** | **Systolic blood pressure (SBP) (mmHg)** | **Diastolic blood pressure (DBP) (mmHg)** | **HDL-cholesterol (mmol /L)** | **Triglycerides (mmol /L)** | **Waist circumference (cm)** | **BMI (kg/m^2^)** | **WHR** |
| --- | --- | --- | --- | --- | --- | --- | --- | --- | --- | --- | --- |
| **α-Carotene** | OR: 0.882 (0.663 – 1.018) ***p* = 0.073** | β = -0.142 (-0.221 ; -0.063) ***p <* 0.001** | β = -0.133 (-0.257 ; -0.009) ***p* = 0.035** | β = -0.113 (-0.206 ; -0.020) ***p* = 0.017** | β = 0.450 (-1.213 ; 2.114) *p* = 0.595 | β = -0.799 (-1.842 ; 0.244) *p* = 0.133 | β = 0.055 (0.019 ; 0.091) ***p* = 0.003** | β = -0.134 (-0.211 ; -0.057) ***p <* 0.001** | β = -1.253 (-2.566 ; 0.059) ***p*** = **0.061** | β = -0.560 (-1.020 ; -0.099) ***p*** = **0.017** | β = -0.010 (-0.020 ; -0.001) ***p*** = **0.029** |
| **β-Carotene** | OR: 0.768 (0.554 – 1.067) *p* = 0.116 | β = -0.162 (-0.281 ; -0.044) ***p*** = **0.007** | β = -0.126 (-0.312 ; -0.060) *p* = 0.185 | β = -0.134 (-0.274 ; 0.006) ***p* = 0.060** | β = 0.057 (-2.439 ; 2.554) *p* = 0.964 | β = -1.493 (-3.057 ; 0.072) ***p* = 0.061** | β = 0.062 (0.008 ; 0.116) ***p* = 0.025** | β = -0.170 (-0.285 ; -0.055) ***p =* 0.004** | β = -1.226 (-3.198 ; 0.745) *p* = 0.223 | β = -0.771 (-1.463 ; -0.080) ***p*** = **0.029** | β = -0.011 (-0.025 ; 0.003) *p* = 0.124 |
| **β-Cryptoxanthin** | OR: 1.13 (0.853 – 1.513) *p* = 0.384 | β = -0.012 (-0.114 ; 0.091) *p* = 0.825 | β = -0.000 (-0.160 ; 0.161) *p* = 0.997 | β = -0.011 (-0.131 ; 0.110) *p* = 0.863 | β = -0.401 (-2.549 ; 1.747) *p* = 0.714 | β = -0.646 (-1.994 ; 0.701) *p* = 0.347 | β = 0.033 (-0.014 ; 0.080) *p* = 0.170 | β = -0.027 (-0.126 ; 0.073) *p* = 0.596 | β = 0.320 (-1.381 ; 2.022) *p* = 0.712 | β = -0.164 (-0.432 ; 0.760) *p* = 0.590 | β = -0.005 (-0.018 ; 0.007) *p* = 0.378 |
| **Total provitamin A** | OR: 0.770 (0.561 – 1.056) *p* = 0.105 | β = -0.162 (-0.276 ; -0.048) ***p* = 0.006** | β = -0.162 (-0.305 ; 0.053) *p* = 0.168 | β = -0.129 (-0.264 ; 0.005) ***p* = 0.060** | β = 0.260 (-2.145 ; 2.665) *p* = 0.832 | β = -1.371 (-2.879 ; 0.136) ***p* = 0.075** | β = 0.063 (0.011 ; 0.116) ***p* = 0.018** | β = -0.168 (-0.279 ; -0.058) ***p* = 0.003** | β = -1.242 (-3.142 ; 0.657) *p* = 0.200 | β = -0.744 (-1.410 ; -0.078) ***p*** = **0.029** | β = -0.011 (-0.024 ; 0.003) *p* = 0.118 |
| **Lycopene** | OR: 0.775 (0.605 – 0.992) ***p* = 0.043** | β = 0.095 (0.001 ; 0.188) ***p* = 0.047** | β = 0.011 (-0.135 ; 0.157) *p* = 0.886 | β = -0.062 (-0.173 ; 0.048) *p* = 0.270 | β = -1.395 (-3.358 ; 0.569) *p* = 0.164 | β = -1.002 (-2.238 ; 0.235) *p* = 0.112 | β = -0.094 (-0.137 ; -0.052) ***p <* 0.001** | β = 0.148 (0.057 ; 0.238) ***p* = 0.001** | β = 1.106 (-0.446 ; 2.657) *p* = 0.162 | β = 0.218 (-0.328 ; 0.764) *p* = 0.434 | β = 0.003 (-0.024 ; 0.003) *p* = 0.118 |
| **Lutein + zeaxanthin** | OR: 1.016 (0.725 – 1.423) *p* = 0.928 | β = -0.107 (-0.228 ; 0.015) ***p* = 0.085** | β = -0.099 (-0.290 ; 0.092) *p* = 0.309 | β = 0.008 (-0.135 ; 0.151) *p* = 0.910 | β = -0.027 (-2.581 ; 2.526) *p* = 0.983 | β = -2.802 (-4.627 ; -0.978) *p* = **0.003** | β = 0.040 (-0.015 ; 0.096) *p* = 0.156 | β = -0.148 (-0.266 ; -0.031) ***p* = 0.014** | β = 0.274 (-1.743 ; 2.291) *p* = 0.790 | β = -0.172 (-0.880 ; -0.536) *p* = 0.634 | β = -0.000 (-0.014 ; 0.014) *p* = 0.993 |
| **Astaxanthin** | OR: 1.184 (0.823 – 1.703) *p* = 0.362 | β = 0.027 (-0.098 ; 0.152) *p* = 0.672 | β = 0.188 (-0.013 ; 0.388) ***p* = 0.067** | β = 0.117 (-0.038 ; 0.273) *p* = 0.139 | β = -0.358 (-3.101 ; 2.384) *p* = 0.798 | β = -0.757 (-2.474 ; 0.960) *p* = 0.387 | β = -0.006 (-0.065 ; 0.054) *p* = 0.855 | β = 0.008 (-0.108 ; 0.124) *p* = 0.893 | β = 2.009 (-0.154 ; 4.172) ***p* = 0.069** | β = 0.244 (-0.308 ; -1.209) *p* = 0.244 | β = 0.018 (0.003 ; 0.033) ***p* = 0.021** |
| **Phytoene** | OR: 1.217 (0.824 – 1.797) *p* = 0.324 | β = 0.014 (-0.125 ; 0.152) *p* = 0.846 | β = -0.049 (-0.266 ; 0.168) *p* = 0.658 | β = -0.159 (-0.322 ; 0.004) ***p =* 0.056** | β = -2.921 (-5.834 ; -0.007) ***p* = 0.049** | β = -2.802 (-4.627 ; -0.978) ***p* = 0.003** | β = -0.034 (-0.098 ; 0.029) *p* = 0.289 | β = -0.035 (-0.169 ; 0.100) *p* = 0.616 | β = 0.629 (-1.676 ; 2.933) *p* = 0.593 | β = 0.339 (-0.470 ; 1.148) *p* = 0.411 | β = -0.639 (-0.020 ; 0.012) *p* = 0.639 |
| **Phytofluene** | OR: 1.141 (0.765 – 1.701) *p* = 0.519 | β = -0.017 (-0.161 ; 0.127) *p* = 0.815 | β = -0.091 (-0.316 ; 0.134) *p* = 0.426 | β = -0.180 (-0.349 ; -0.010) ***p* = 0.037** | β = -3.686 (-6.698 ; -0.675) ***p* = 0.016** | β = -3.209 (-5.096 ; -1.321) ***p* = 0.001** | β = -0.020 (-0.086 ; 0.046) *p* = 0.549 | β = -0.061 (-0.201 ; 0.079) *p* = 0.392 | β = 0.525 (-1.862 ; 2.911) *p* = 0.666 | β = 0.355 (-0.483 ; 1.193) *p* = 0.406 | β = -0.006 (-0.023 ; 0.011) *p* = 0.484 |
| **Neoxanthin** | OR: 1.614 (1.008 – 2.583) ***p* = 0.046** | β = 0.015 (-0.153 ; 0.183) *p* = 0.861 | β = 0.142 (-0.120 ; -0.405) *p* = 0.287 | β = 0.121 (-0.076 ; -0.319) *p* = 0.227 | β = 4.456 (0.941 ; 7.970) ***p* = 0.013** | β = -0.164 (-2.374 ; .046) *p* = 0.884 | β = 0.022 (-0.055 ; 0.099) *p* = 0.570 | β = -0.097 (-0.260 ; 0.066) *p* = 0.245 | β = 1.267 (-1.514 ; 4.048) *p* = 0.372 | β = -0.165 (-1.142 ; 0.812) *p* = 0.741 | β = 0.011 (-0.009 ; 0.031) ***p* = 0.011** |
| **Violaxanthin** | OR: 0.987 (0.655 – 1.489) *p* = 0.952 | β = -0.077 (-0.225 ; 0.072) *p* = 0.311 | β = -0.097 (-0.329 ; 0.134) *p* = 0.410 | β = 0.006 (-0.168 ; -0.181) *p* = 0.942 | β = 0.272 (-2.841 ; 3.386) *p* = 0.864 | β = -1.809 (-3.760 ; 0.142) ***p* = 0.069** | β = 0.039 (-0.029 ; 0.107) *p* = 0.262 | β = -0.130 (-0.274 ; 0.014) ***p =* 0.077** | β = -0.840 (-3.299 ; 1.620) *p* = 0.503 | β = 0.122 (-1.544 ; 0.182) *p* = 0.122 | β = -0.010 (-0.028 ; 0.007) *p* = 0.258 |
| **Phytoene+ Phtofluene** | OR: 1.205 (0.817 – 1.779) *p* = 0.347 | β = 0.011 (-0.127 ; 0.150) *p* = 0.874 | β = -0.051 (-0.268 ; 0.166) *p* = 0.644 | β = -0.158 (-0.321 ; 0.005) ***p* = 0.057** | β = -2.836 (-5.744 ; 0.071) ***p =* 0.056** | β = -2.772 (-4.593 ; -0.951) ***p* = 0.003** | β = -0.033 (-0.096 ; 0.031) *p* = 0.311 | β = -0.036 (-0.171 ; 0.098) *p* = 0.597 | β = 0.598 (-1.702 ; 2.897) *p* = 0.610 | β = 0.316 (-0.443 ; 1.124) *p* = 0.443 | β = -0.004 (0.020 ; 0.012) *p* = 0.639 |
| **Epoxycarotenoids** | OR: 1.111 (0.720 – 1.715) *p* = 0.633 | β = -0.058 (-0.214 ; 0.098) *p* = 0.464 | β = -0.045 (-0.289 ; -0.200) *p* = 0.720 | β = 0.036 (-0.147 ; 0.220) *p* = 0.697 | β = 1.280 (-1.999 ; 4.558) *p* = 0.444 | β = -1.495 (-3.551 ; 0.561) *p* = 0.154 | β = 0.037 (-0.035 ; 0.109) *p* = 0.310 | β = -0.127 (-0.279 ; 0.024) ***p* = 0.099** | β = -0.389 (-2.979 ; 2.201) *p* = 0.768 | β = -0.596 (-1.505 ; 0.314) *p* = 0.199 | β = -0.006 (-0.024 ; 0.013) *p* = 0.548 |
| **Total non-provitamin A** | OR: 0.899 (0.542 – 1.491) *p* = 0.680 | β = 0.063 (-0.120 ; 0.245) *p* = 0.501 | β = -0.045 (-0.331 ; 0.240) *p* = 0.755 | β = -0.139 (-0.354 ; 0.076) *p* = 0.204 | β = -3.100 (-6.932 ; 0.733) *p =* 0.113 | β = -3.033 (-5.435 ; -0.631) ***p* = 0.013** | β = -0.113 (-0.196 ; -0.029) ***p* = 0.008** | β = 0.056 (-0.121 ; 0.233) *p* = 0.536 | β = 1.986 (-1.043 ; 5.015) *p* = 0.199 | β = 0.378 (-0.686 ; 1.442) *p* = 0.486 | β = 0.003 (-0.019 ; 0.024) *p* = 0.814 |
| **Total carotenoids** | OR: 0.756 (0.475 – 1.202) *p* = 0.237 | β = -0.069 (-0.237 ; 0.098) *p* = 0.415 | β = -0.060 (-0.322 ; 0.202) *p* = 0.653 | β = -0.161 (-0.357 ; 0.036) *p* = 0.109 | β = -1.014 (-4.526 ; 2.498) *p =* 0.571 | β = -2.492 (-4.692 ; -0.293) ***p* = 0.026** | β = -0.009 (-0.085 ; 0.068) *p* = 0.827 | β = -0.065 (-0.227 ; 0.098) *p* = 0.436 | β = 0.107 (-2.669 ; 2.882) *p* = 0.940 | β = -0.404 (-1.378 ; 0.570) *p* = 0.416 | β = -0.006 (-0.026 ; 0.014) *p* = 0.566 |
| **Fruits** | OR: 1.741 (1.201 – 2.523) ***p* = 0.003** | β = -0.016 (-0.143 ; 0.110) *p* = *0.802* | β = 0.153 (-0.044 ; 0.350) *p* = 0.128 | β = 0.019 (-0.130 ; 0.168) *p* = 0.803 | β = 1.424 (-1.231 ; 4.080) *p* = 0.293 | β = 0.109 (1.557 ; 1.775) *p* = 0.898 | β = 0.019 (-0.039 ; 0.077) *p* = 0.519 | β = -0.085 (-0.208 ; 0.038) *p* = *0.175* | β = 1.400 (-0.695 ; 3.495) *p* = 0.190 | β = 0.329 (-0.408 ; 1.066) *p* = 0.382 | β = 0.006 (-0.009 ; 0.021) *p* = 0.429 |
| **Vegetables** | OR: 0.812 (0.533 – 1.237) *p* = 0.332 | β = -0.163 (-0.316 ; -0.011) ***p* = 0.035** | β = -0.213 (-0.452 ; 0.026) ***p* = 0.081** | β = -0.136 (-0.316 ; 0.044) *p* = 0.137 | β = -3.119 (-6.323 ; 0.085) ***p =* 0.056** | β = -3.884 (-5.884 ; -1.883) ***p <* 0.001** | β = 0.061 (-0.009 ; 0.131) ***p* = 0.085** | β = -0.174 (-0.321 ; 0.026) ***p* = 0.021** | β = -2.234 (-4.765 ; 0.297) ***p* = 0.084** | β = -0.959 (-1.847 ; -0.070) ***p*** = **0.035** | β = -0.021 (-0.039 ; -0.003) *p* = 0.020 |
| **Fruits+Vegetables** | OR: 1.475 (0.912 – 2.387) *p* = 0.113 | β = -0.119 (-0.290 ; 0.051) *p* = 0.171 | β = 0.069 (-0.238 ; 0.296) *p* = 0.830 | β = -0.053 (-0.254 ; 0.148) *p* = 0.605 | β = -0.485 (-4.073 ; 3.103) *p =* 0.791 | β = -2.247 (-4.496 ; 0.001) ***p* = 0.050** | β = 0.066 (-0.012 ; 0.144) ***p* = 0.099** | β = -0.177 (-0.343 ; 0.011) ***p* = 0.036** | β = -0.077 (-2.911 ; 2.757) *p* = 0.958 | β = -0.259 (-1.254 ; 0.737) *p* = 0.611 | β = -0.008 (-0.028 ; 0.012) *p* = 0.441 |

ORISCAV: Observation of Cardiovascular Risk Factors.

Metabolic syndrome (MetS) was diagnosed with the NCEP-ATP III criteria.

siMetS score and siMetS risk score are continuous metabolic syndrome (MetS) scores used to quantify metabolic status and metabolic risk of cardio/cerebrovascular events, respectively.

No confounder was considered for this crude model (model 1).

All predictor variables were log-transformed before multivariable regression models.

BMI: Body mass index (kg/m^2^). WHR: Waist-hip ratio.

Among anthropometric measurements including BMI and WHR, only waist circumference is considered as a component of MetS.

Data for logistic regression (only for MetS ATP III) are expressed as odds ratio (OR) with its 95% confidence interval (95% CI).

Data for linear regression are expressed as beta (β) regression coefficient with its 95% confidence interval (95% CI).

Total pro-vitamin A species corresponds to the sum of α-carotene, β-carotene and β-cryptoxanthin.

Epoxycarotenoids is the sum of neoxanthin and violaxanthin.

Phytoene+phtofluene: the sum of phytoene and phtofluene represents colorless carotenoids.

Total non-provitamin A species corresponds to the sum of lycopene, lutein + zeaxanthin, astaxanthin, phytoene, phtofluene, neoxanthin and violaxanthin.

Total carotenoids corresponds to the sum of the total pro-vitamin A carotenoids and total non-provitamin A carotenoids.

Fruits+Vegetables corresponds to the combined intake of fruits and vegetables.

Significant (*p* < 0.05) and tendency (*p* < 0.1) p-values are given in bold. *P*-values in blue ink indicate a tendency, i.e. *p*-value>0.05 but <0.1.

**Supplementary table 2.** Adjusted logistic and linear regression models for age and gender associating carotenoid, fruit and vegetable intake with metabolic syndrome (MetS), its scores, components and additional anthropometric measurements of Luxembourgish participants (n=1346) of the ORISCAV-2 study**.**

| **Carotenoid intake**  **(µg /day)** | **MetS (APT III)** | **siMetS score (metabolic status)** | **siMetS risk score (metabolic risk)*** | **Fasting blood glucose (FBG) (mmol /L)** | **Systolic blood pressure (SBP) (mmHg)** | **Diastolic blood pressure (DBP) (mmHg)** | **HDL-cholesterol (mmol /L)** | **Triglycerides (mmol /L)** | **Waist circumference (cm)** | **BMI (kg/m^2^)** | **WHR** |
| --- | --- | --- | --- | --- | --- | --- | --- | --- | --- | --- | --- |
| **α-Carotene** | OR: 0.721 (0.569 – 0.912) ***p* = 0.006** | β = -0.140 (-0.214 ; -0.065) ***p <* 0.001** | β = -0.133 (-0.257 ; -0.009) ***p* = 0.035** | β = -0.124 (-0.213 ; -0.035) ***p* = 0.006** | β = 0.301 (-1.133 ; 1.734) *p* = 0.681 | β = -0.805 (-1.798 ; 0.188) *p* = 0.112 | β = 0.034 (0.002 ; 0.066) ***p* = 0.039** | β = -0.122 (-0.197 ; -0.048) ***p* = 0.001** | β = -1.034 (-2.178 ; 0.110) ***p* = 0.076** | β = -0.549 (-0.995 ; -0.103) ***p* = 0.016** | β = -0.008 (-0.014 ; -0.001) ***p* = 0.028** |
| **β-Carotene** | OR: 0.624 (0.433 – 0.900) ***p* = 0.012** | β = -0.158 (-0.270 ; -0.046) ***p* = 0.006** | β = -0.126 (-0.312 ; -0.060) *p* = 0.185 | β = -0.155 (-0.288 ; -0.021) ***p* = 0.023** | β = -0.253 (-2.408 ; 1.902) *p* = 0.818 | β = -1.522 (-3.014 ; -0.031) ***p* = 0.045** | β = 0.024 (-0.025 ; 0.027) *p* = 0.336 | β = -0.150 (-0.262 ; -0.038) ***p* = 0.009** | β = -0.848 (-2.569 ; 0.874) *p* = 0.334 | β = -0.757 (-1.427 ; -0.086) ***p* = 0.027** | β = -0.006 (-0.016 ; 0.004) *p* = 0.234 |
| **β-Cryptoxanthin** | OR: 1.025 (0.747 – 1.407) *p* = 0.879 | β = -0.013 (-0.111 ; -0.084) *p* = 0.785 | β = -0.000 (-0.160 ; 0.161) *p* = 0.997 | β = -0.037 (-0.153 ; -0.078) *p* = 0.528 | β = -0.986 (-2.843 ; 0.871) *p* = 0.298 | β = -0.776 (-2.063 ; 0.511) *p* = 0.237 | β = -0.002 (-0.044 ; 0.040) *p* = 0.930 | β = -0.011 (-0.108 ; 0.085) *p* = 0.818 | β = 0.509 (-0.979 ; 1.998) *p* = 0.502 | β = 0.149 (-0.430 ; 0.728) *p* = 0.613 | β = -0.003 (-0.011 ; 0.006) *p* = 0.563 |
| **Total provitamin A** | OR: 0.624 (0.439 – 0.888) ***p* = 0.009** | β = -0.160 (-0.268 ; -0.052) ***p* = 0.004** | β = -0.162 (-0.305 ; 0.053) *p* = 0.168 | β = -0.155 (-0.279 ; -0.022) ***p* = 0.022** | β = -0.084 (-2.160 ; 1.992) *p* = 0.937 | β = -1.418 (-2.855 ; 0.019) ***p* = 0.053** | β = 0.027 (-0.020 ; 0.074) *p* = 0.256 | β = -0.150 (-0.258 ; -0.042) ***p* = 0.006** | β = -0.917 (-2.575 ; 0.742) *p* = 0.278 | β = -0.737 (-1.382 ; -0.091) ***p* = 0.025** | β = -0.007 (-0.016 ; 0.003) *p* = 0.193 |
| **Lycopene** | OR: 0.984 (0.744 – 1.301) *p* = 0.909 | β = 0.106 (0.016 ; -0.196) ***p* = 0.022** | β = 0.011 (-0.135 ; 0.157) *p* = 0.886 | β = -0.015 (-0.123 ; 0.093) *p* = 0.788 | β = -0.350 (-2.080 ; 1.381) *p* = 0.692 | β = -0.797 (-2.001 ; 0.408) *p* = 0.195 | β = -0.037 (-0.076 ; 0.002) ***p* = 0.065** | β = 0.129 (0.038 ; 0.219) ***p* = 0.005** | β = 0.889 (-0.497 ; 2.275) *p* = 0.209 | β = 0.270 (-0.271 ; 0.811) *p* = 0.327 | β = -0.001 (-0.009 ; 0.007) *p* = 0.786 |
| **Lutein + zeaxanthin** | OR: 0.855 (0.589 – 1.239) *p* = 0.407 | β = -0.140 (-0.254 ; -0.025) ***p* = 0.017** | β = -0.099 (-0.290 ; 0.092) *p* = 0.309 | β = -0.043 (-0.179 ; 0.094) *p* = 0.541 | β = -1.356 (-3.555 ; 0.843) *p* = 0.227 | β = -1.337 (-2.861 ; 0.187) ***p* = 0.085** | β = 0.026 (-0.024 ; 0.075) *p* = 0.308 | β = -0.158 (-0.272 ; -0.043) ***p*** **= 0.007** | β = -0.264 (-2.023 ; 1.494) *p* = 0.768 | β = -0.315 (-1.001 ; 0.371) *p* = 0.368 | β = -0.005 (-0.015 ; 0.006) *p* = 0.349 |
| **Astaxanthin** | OR: 0.968 (0.650 – 1.441 *p* = 0.871 | β = -0.044 (-0.162 ; 0.074) *p* = 0.465 | β = 0.188 (-0.013 ; 0.388) ***p* = 0.067** | β = 0.040 (-0.109 ; 0.189) *p* = 0.596 | β = -2.679 (-5.043 ; -0.315) ***p* = 0.026** | β = -1.651 (-3.287 ; -0.015) ***p* = 0.048** | β = 0.018 (-0.035 ; 0.071) *p* = 0.508 | β = -0.039 (-0.151 ; 0.073) *p* = 0.498 | β = 0.392 (-1.510 ; 2.295) *p* = 0.686 | β = 0.144 (-0.593 ; 0.881) *p* = 0.702 | β = 0.002 (-0.009 ; 0.013) *p* = 0.708 |
| **Phytoene** | OR: 1.266 (0.823 – 1.947) *p* = 0.283 | β = 0.045 (-0.086 ; 0.175) *p* = 0.505 | β = -0.049 (-0.266 ; 0.168) *p* = 0.658 | β = -0.153 (-0.309 ; 0.003) ***p* = 0.055** | β = -2.476 (-4.988 ; 0.315) ***p* = 0.053** | β = -2.536 (-4.275 ; -0.798) ***p* = 0.004** | β = -0.082 (-0.139 ; -0.026) ***p* = 0.004** | β = 0.004 (-0.127 ; 0.135) *p* = 0.953 | β = 1.597 (-0.411 ; 3.604) *p* = 0.119 | β = 0.475 (-0.308 ; 1.259) *p* = 0.234 | β = 0.007 (-0.005 ; 0.018) *p* = 0.282 |
| **Phytofluene** | OR: 1.170 (0.753 – 1.820) *p* = 0.485 | β = 0.018 (-0.118 ; 0.154) *p* = 0.793 | β = -0.091 (-0.316 ; 0.134) *p* = 0.426 | β = -0.172 (-0.334 ; -0.011) ***p* = 0.037** | β = -3.183 (-5.782 ; -0.585) ***p* = 0.016** | β = -2.907 (-4.708 ; -1.107) ***p* = 0.002** | β = -0.076 (-0.134 ; -0.017) ***p* = 0.011** | β = -0.017 (-0.152 ; 0.119) *p* = 0.808 | β = 1.650 (-0.432 ; 3.731) *p* = 0.120 | β = 0.515 (-0.297 ; 1.327) *p* = 0.214 | β = 0.006 (-0.006 ; 0.018) *p* = 0.337 |
| **Neoxanthin** | OR: 0.943 (0.557 – 2.563) *p* = 0.828 | β = -0.085 (-0.244 ; 0.075) *p* = 0.298 | β = 0.142 (-0.120 ; -0.405) *p* = 0.287 | β = -0.025 (-0.216 ; 0.166) *p* = 0.797 | β = 0.507 (-2.562 ; 3.576) *p* = 0.746 | β = -1.563 (-3.689 ; 0.563) *p* = 0.150 | β = -0.008 (-0.076 ; 0.061) *p* = 0.829 | β = -0.134 (-0.294 ; 0.025) ***p* = 0.098** | β = -0.581 (-3.032 ; 1.871) *p* = 0.642 | β = -0.630 (-1.586 ; 0.326) *p* = 0.196 | β = -0.005 (-0.019 ; -0.010) *p* = 0.523 |
| **Violaxanthin** | OR: 0.788 (0.497 – 1.249) *p* = 0.311 | β = -0.083 (-0.223 ; 0.057) *p* = 0.244 | β = -0.097 (-0.329 ; 0.134) *p* = 0.410 | β = -0.035 (-0.203 ; 0.132) *p* = 0.678 | β = -0.650 (-3.343 ; 2.043) *p* = 0.636 | β = -2.041 (-3.905 ; -0.177) ***p* = 0.032** | β = -0.012 (-0.072 ; 0.049) *p* = 0.703 | β = -0.110 (-0.250 ; 0.030) *p* = 0.124 | β = -0.636 (-2.787 ; 1.516) *p* = 0.562 | β = -0.726 (-1.565 ; 0.112) ***p* = 0.090** | β = -0.007 (-0.019 ; -0.006) *p* = 0.317 |
| **Phytoene+ Phtofluene** | OR: 1.249 (0.813 – 1.920) *p* = 0.309 | β = 0.042 (-0.089 ; 0.172) *p* = 0.533 | β = -0.051 (-0.268 ; 0.166) *p* = 0.644 | β = -0.152 (-0.308 ; 0.003) ***p* = 0.055** | β = -2.399 (-4.906 ; 0.108) ***p* = 0.061** | β = -2.510 (-4.245 ; -0.775) ***p* = 0.005** | β = -0.080 (-0.136 ; -0.024) ***p* = 0.005** | β = 0.002 (-0.129 ; 0.132) *p* = 0.979 | β = 1.555 (-0.499 ; 3.558) *p* = 0.128 | β = 0.451 (-0.331 ; 1.233) *p* = 0.258 | β = 0.006 (-0.005 ; 0.018) *p* = 0.290 |
| **Epoxycarotenoids** | OR: 0.817 (0.502 – 1.329) *p* = 0.415 | β = -0.087 (-0.235 ; 0.061) *p* = 0.249 | β = -0.045 (-0.289 ; -0.200) *p* = 0.720 | β = -0.032 (-0.208 ; 0.145) *p* = 0.724 | β = -0.393 (-3.234 ; 2.447) *p* = 0.786 | β = -2.009 (-3.976 ; -0.043) ***p* = 0.045** | β = -0.011 (-0.075 ; 0.052) *p* = 0.703 | β = -0.120 (-0.267 ; 0.028) *p* = 0.112 | β = -0.652 (-2.921 ; 1.618) *p* = 0.573 | β = -0.740 (-1.625 ; 0.144) *p* = 0.101 | β = -0.006 (-0.020 ; 0.007) *p* = 0.347 |
| **Total non-provitamin A** | OR: 1.113 (0.637 – 1.942) *p* = 0.707 | β = 0.066 (-0.106 ; 0.237) ***p* = 0.066** | β = -0.045 (-0.331 ; 0.240) *p* = 0.755 | β = -0.116 (-0.320 ; 0.089) *p* = 0.269 | β = -2.570 (-5.866 ; 0.727) *p* = 0.127 | β = -2.923 (-5.205 ; -0.641) ***p* = 0.012** | β = -0.083 (-0.157 ; -0.010) ***p* = 0.027** | β = 0.043 (-0.128 ; 0.215) *p* = 0.619 | β = 1.871 (-0.763 ; 4.505) *p* = 0.164 | β = 0.400 (-0.629 ; 1.428) *p* = 0.446 | β = 0.001 (-0.015 ; 0.016) *p* = 0.949 |
| **Total carotenoids** | OR: 0.703 (0.423 – 1.170) *p* = 0.175 | β = -0.070 (-0.227 ; 0.087) *p* = 0.379 | β = -0.060 (-0.322 ; 0.202) *p* = 0.653 | β = -0.168 (-0.355 ; 0.019) ***p* = 0.078** | β = -1.160 (-4.176 ; 1.855) *p* = 0.451 | β = -2.521 (-4.607 ; -0.434) ***p* = 0.018** | β = -0.019 (-0.086 ; 0.049) *p* = 0.591 | β = -0.060 (-0.217 ; 0.097) *p* = 0.455 | β = 1.171 (-2.240 ; 2.582)*p* = 0.890 | β = -0.407 (-1.347 ; 0.533) *p* = 0.396 | β = -0.005 (-0.019 ; 0.009) *p* = 0.507 |
| **Fruits** | OR: 1.265 (0.844 – 1.849) *p* = 0.255 | β = -0.078 (-0.198 ; 0.042) *p* = 0.203 | β = 0.153 (-0.044 ; 0.350) *p* = 0.128 | β = -0.079 (-0.223 ; 0.064) *p* = 0.280 | β = -1.218 (-3.524 ; 1.089) *p* = 0.301 | β = -0.810 (-2.408 ; 0.789) *p* = 0.321 | β = -0.007 (-0.059 ; 0.044) *p* = 0.781 | β = -0.105 (-0.225 ; 0.015) ***p* = 0.087** | β = 0.314 (-1.530 ; 2.158) *p* = 0.738 | β = 0.049 (-0.672 ; 0.769) *p* = 0.894 | β = -0.003 (-0.014 ; 0.008) *p* = 0.550 |
| **Vegetables** | OR: 0.709 (0.441 – 1.142) *p* = 0.158 | β = -0.122 (-0.266 ; 0.022) ***p* = 0.098** | β = -0.213 (-0.452 ; 0.026) ***p* = 0.081** | β = -0.130 (-0.303 ; 0.042) *p* = 0.139 | β = -2.560 (-5.334 ; 0.215) ***p* = 0.071** | β = -3.634 (-5.461 ; -1.632) ***p <* 0.001** | β = -0.012 (-0.074 ; 0.051) *p* = 0.711 | β = -0.119 (-0.263 ; 0.025) *p* = 0.105 | β = -0.843 (-3.061 ; 1.375) *p* = 0.456 | β = -0.780 (-1.645 ; 0.085) ***p* = 0.077** | β = -0.007 (-0.019 ; 0.007) *p* = 0.367 |
| **Fruits+Vegetables** | OR: 0.991 (0.577 – 1.704) *p* = 0.975 | β = -0.160 (-0.322 ; 0.003) ***p* = 0.054** | β = 0.069 (-0.238 ; 0.296) *p* = 0.830 | β = -0.150 (-0.344 ; 0.044) *p* = 0.130 | β = -2.832 (-5.954 ; 0.290) ***p* = 0.075** | β = -2.979 (-5.139 ; -0.818) ***p* = 0.007** | β = -0.002 (-0.072 ; 0.068) *p* = 0.963 | β = -0.167 (-0.329 ; -0.005) ***p* = 0.044** | β = -0.401 (-2.897 ; 2.095) *p* = 0.753 | β = -0.447 (-1.421 ; -0.527) *p* = 0.368 | β = -0.009 (-0.024 ; 0.006) *p* = 0.244 |

ORISCAV: Observation of Cardiovascular Risk Factors.

Metabolic syndrome (MetS) was diagnosed with the NCEP-ATP III criteria.

siMetS score and siMetS risk score are continuous metabolic syndrome (MetS) scores used to quantify metabolic status and metabolic risk of cardio/cerebrovascular events, respectively.

The confounders of this model (model 2) are: age and sex.

*Regression analyses for siMetS risk score did not include sex and age as confounders, as they were included in the formula of this score.

All predictor variables were log-transformed before multivariable regression models.

BMI: Body mass index (kg/m^2^). WHR: Waist-hip ratio.

Among anthropometric measurements including BMI and WHR, only waist circumference is considered as a component of MetS.

Data for logistic regression (only for MetS ATP III) are expressed as odds ratio (OR) with its 95% confidence interval (95% CI).

Data for linear regression are expressed as beta (β) regression coefficient with its 95% confidence interval (95% CI).

Total pro-vitamin A species corresponds to the sum of α-carotene, β-carotene and β-cryptoxanthin.

Epoxycarotenoids is the sum of neoxanthin and violaxanthin.

Phytoene+ phtofluene: the sum of phytoene and phtofluene represents colorless carotenoids.

Total non-provitamin A species corresponds to the sum of lycopene, lutein + zeaxanthin, astaxanthin, phytoene, phtofluene, neoxanthin and violaxanthin.

Total carotenoids corresponds to the sum of the total pro-vitamin A carotenoids and total non-provitamin A carotenoids.

Fruits+Vegetables corresponds to the combined intake of fruits and vegetables.

Significant (*p* < 0.05) and tendency (*p* < 0.1) p-values are given in bold. *P*-values in blue ink indicate a tendency, i.e. *p*-value>0.05 but <0.1.

**Supplementary table 3.** Adjusted logistic and linear regression models associating carotenoid intake with metabolic syndrome (MetS), its scores, components and additional anthropometric measurements of Luxembourgish participants (n=1346) of the ORISCAV-2 study.

| **Carotenoid intake**  **(µg /day)** | **MetS (APT III)** | **siMetS score (metabolic status)** | **siMetS risk score (metabolic risk)*** | **Fasting blood glucose (FBG) (mmol /L)** | **Systolic blood pressure (SBP) (mmHg)** | **Diastolic blood pressure (DBP) (mmHg)** | **HDL-cholesterol (mmol /L)** | **Triglycerides (mmol /L)** | **Waist circumference (cm)** | **BMI (kg/m^2^)** | **WHR** |
| --- | --- | --- | --- | --- | --- | --- | --- | --- | --- | --- | --- |
| **α-Carotene** | OR: 0.718 (0.561 – 0.920) ***p* = 0.009** | β = -0.151 (-0.226 ; -0.077) ***p < 0.001*** | β = -0.232 (-0.349 ; -0.116) ***p < 0.001*** | β = -0.136 (-2.226 ; -0.046) ***p* = 0.003** | β = 0.346 (-1.116 ; 1.807) *p* = 0.643 | β = -0.725 (-1.733 ; 0.283) *p* = 0.159 | β = 0.038 (0.006 ; 0.070) ***p* = 0.022** | β = -0.127 (-0.201 ; -0.052) ***p* = 0.001** | β = -1.394 (-2.548 ; -0.241) ***p* = 0.018** | β = **-**0.619 (-1.071 ; -0.168) ***p* = 0.007** | β = **-**0.009 (-0.016 ; -0.002) ***p* = 0.012** |
| **β-Carotene** | OR: 0.599 (0.405 – 0.886) ***p* = 0.010** | β = -0.189 (-0.303 ; -0.076) ***p* = 0.001** | β = -0.302 (-0.480 ; -0.124) ***p =* 0.001** | β = -0.186 (-0.324 ; -0.048) ***p* = 0.070** | β = -0.179 (-2.414 ; 2.056) *p* = 0.875 | β = -1.533 (-3.074 ; 0.007) ***p* = 0.051** | β = 0.032 (-0.017 ; 0.081) *p* = 0.203 | β = -0.166 (-0.280 ; -0.051) ***p* = 0.005** | β = -1.731 (-3.497 ; 0.036) ***p* = 0.055** | β = -0.961 (-1.652 ; -0.271) ***p* = 0.006** | β = -0.009 (-0.019 ; 0.002) *p* = 0.102 |
| **β-Cryptoxanthin** | OR: 1.017 (0.729 – 1.419) *p* = 0.921 | β = -0.023 (-0.122 ; 0.076) *p* = 0.647 | β = -0.091 (-0.245 ; 0.063) *p =* 0.245 | β = -0.054 (-0.173 ; 0.066) *p* = 0.377 | β = -0.965 (-2.892 ; 0.963) *p* = 0.326 | β = -0.913 (-2.244 ; 0.417) *p* = 0.178 | β = -0.0004 (-0.043 ; 0.042) *p* = 0.983 | β = -0.008 (-0.107 ; 0.090) *p* = 0.867 | β = -0.209 (-1.740 ; 1.322) *p* = 0.789 | β = -0.009 (-0.606 ; 0.589) *p* = 0.978 | β = -0.004 (-0.013 ; 0.005) *p* = 0.415 |
| **Total provitamin A** | OR: 0.601 (0.413 – 0.876) ***p* = 0.008** | β = -0.187 (-0.297 ; -0.078) ***p* = 0.001** | β = -0.295 (-0.466 ; -0.123) ***p < 0.001*** | β = -0.178 (-0.311 ; -0.046) ***p* = 0.009** | β = -0.006 (-2.153 ; 2.142) *p* = 0.996 | β = -1.403 (-2.883 ; 0.078) ***p* = 0.063** | β = 0.035 (-0.013 ; 0.082) *p* = 0.150 | β = -0.163 (-0.273 ; -0.053) ***p* = 0.004** | β = -1.731 (-3.429 ; -0.034) ***p* = 0.046** | β = **-**0.925 (-1.588 ; -0.261) ***p* = 0.006** | β = -0.009 (-0.019 ; 0.001) ***p* = 0.085** |
| **Lycopene** | OR: 0.959 (0.711 – 1.296) *p* = 0.787 | β = 0.085 (-0.010 ; -0.179) ***p* = 0.079** | β = -0.049 (-0.197 ; 0.098) *p =* 0.512 | β = -0.051 (-0.166 ; 0.064) *p* = 0.383 | β = -0.264 (-2.107 ; 1.578) *p* = 0.778 | β = -0.854 (-2.130 ; 0.422) *p* = 0.190 | β = -0.034 (-0.075 ; 0.007) *p* = 0.100 | β = 0.117 (0.022 ; 0.212) ***p* = 0.015** | β = -0.076 (-1.385 ; 1.538) *p* = 0.918 | β = 0.155 (-0.417 ; 0.728) *p* = 0.594 | β = -0.004 (-0.013 ; 0.004) *p* = 0.339 |
| **Lutein + zeaxanthin** | OR: 0.833 (0.560 – 1.239) *p* = 0.367 | β = -0.175 (-0.292 ; -0.059) ***p* = 0.003** | β = -0.258 (-0.441 ; -0.076) ***p =* 0.006** | β = -0.068 (-0.209 ; 0.074) *p* = 0.348 | β = -1.367 (-3.649 ; 0.916) *p* = 0.240 | β = -1.496 (-3.070 ; 0.078) ***p* = 0.062** | β = 0.035 (-0.016 ; 0.085) *p* = 0.175 | β = -0.181 (-0.297 ; -0.064) ***p* = 0.002** | β = -1.190 (-2.995 ; 0.616) *p* = 0.196 | β = -0.517 (-1.224 ; 0.190) *p* = 0.152 | β = -0.007 (-0.018 ; 0.004) *p* = 0.189 |
| **Astaxanthin** | OR: 0.930 (0.611 – 1.413 *p* = 0.733 | β = -0.044 (-0.162 ; 0.074) *p* = 0.465 | β = 0.030 (-0.162 ; 0.223) *p =* 0.759 | β = 0.013 (-0.139 ; 0.166) *p* = 0.863 | β = -2.671 (-5.098 ; -0.243) ***p* = 0.031** | β = -1.671 (-3.342 ; 0.001) ***p* = 0.050** | β = 0.021 (-0.033 ; 0.075) *p* = 0.443 | β = -0.054 (-0.168 ; 0.059) *p* = 0.349 | β = -0.322 (-2.255 ; 1.611) *p* = 0.744 | β = 0.020 (-0.732 ; 0.772) *p* = 0.959 | β = 0.000 (-0.011 ; 0.012) *p* = 0.964 |
| **Phytoene** | OR: 1.216 (0.764 – 1.935) *p* = 0.409 | β = 0.014 (-0.122 ; 0.150) *p* = 0.841 | β = -0.227 (-0.439 ; -0.015) ***p* = 0.036** | β = -0.209 (-0.374 ; -0.044) ***p* = 0.013** | β = -2.623 (-5.286 ; 0.041) ***p* = 0.054** | β = -2.877 (-4.711 ; -1.043) ***p* = 0.002** | β = -0.083 (-0.142 ; -0.024) ***p* = 0.006** | β = -0.011 (-0.147 ; 0.126) *p* = 0.879 | β = 0.366 (-1.744 ; 2.476) *p* = 0.734 | β = 0.242 (-0.584 ; 1.068) *p* = 0.566 | β = 0.004 (-0.008 ; 0.017) *p* = 0.524 |
| **Phtofluene** | OR: 1.108 (0.685 – 1.793) *p* = 0.676 | β = -0.017 (-0.159 ; 0.124) *p* = 0.811 | β = -0.267 (-0.487 ; -0.048) ***p =* 0.017** | β = -0.234 (-0.405 ; -0.063) ***p* = 0.007** | β = -3.371 (-6.130 ; -0.612) ***p* = 0.017** | β = -3.334 (-5.236 ; -1.432) ***p* = 0.001** | β = -0.075 (-0.136 ; -0.014) ***p* = 0.016** | β = -0.034 (-0.176 ; 0.108) *p* = 0.641 | β = 0.328 (-1.863 ; 2.518) *p* = 0.769 | β = 0.252 (-0.605 ; 1.110) *p* = 0.564 | β = 0.003 (-0.010 ; 0.016) *p* = 0.607 |
| **Neoxanthin** | OR: 0.864 (0.489 – 1.528) *p* = 0.615 | β = -0.125 (-0.291 ; 0.042) *p* = 0.142 | β = -0.275 (-0.534 ; -0.015) ***p =* 0.038** | β = -0.066 (-0.268 ; 0.137) *p* = 0.524 | β = 0.816 (-2.447 ; 4.080) *p* = 0.624 | β = -1.465 (-3.717 ; 0.786) *p* = 0.202 | β = 0.001 (-0.071 ; -0.073) *p* = 0.983 | β = -0.158 (-0.325 ; 0.009) ***p* = 0.064** | β = -2.164 (-4.743 ; 0.415) ***p* = 0.099** | β = -0.983 (-1.993 ; 0.027) *p* = **0.056** | β = -0.009 (-0.024 ; 0.006) *p* = 0.249 |
| **Violaxanthin** | OR: 0.757 (0.463 – 1.238) *p* = 0.267 | β = -0.105 (-0.248 ; 0.039) *p* = 0.152 | β = -0.311 (-0.535 ; -0.088) ***p =* 0.006** | β = -0.064 (-0.238 ; 0.110) *p* = 0.470 | β = -0.527 (-3.339 ; 2.284) *p* = 0.713 | β = -2.138 (-4.075 ; -0.200) ***p* = 0.031** | β = -0.012 (-0.074 ; 0.050) *p* = 0.710 | β = -0.122 (-0.266 ; 0.022) ***p* = 0.097** | β = -1.684 (-3.907 ; 0.539) *p* = 0.138 | β = -0.952 (-1.822 ; -0.083) ***p* = 0.032** | β = -0.009 (-0.022 ; 0.004) *p* = 0.191 |
| **Phytoene+ Phtofluene** | OR: 1.200 (0.755 – 1.906) *p* = 0.441 | β = 0.011 (-0.125 ; 0.147) *p* = 0.875 | β = -0.229 (-0.441 ; -0.018) ***p =* 0.033** | β = -0.208 (-0.373 ; -0.044) ***p* = 0.013** | β = -0.264 (-2.107 ; 1.578) *p* = 0.778 | β = -2.842 (-4.672 ; -1.012) ***p* = 0.002** | β = -0.081 (-0.139 ; -0.022) ***p* = 0.007** | β = -0.013 (-0.149 ; 0.123) *p* = 0.852 | β = 0.329 (-1.776 ; 2.434) *p* = 0.759 | β = 0.217 (-0.607 ; 1.041) *p* = 0.605 | β = 0.004 (-0.009 ; 0.016) *p* = 0.534 |
| **Epoxycarotenoids** | OR: 0.775 (0.460 – 1.307) *p* = 0.339 | β = -0.114 (-0.266 ; 0.039) *p* = 0.143 | β = -0.315 (-0.551 ; -0.078) ***p =* 0.009** | β = -0.064 (-0.249 ; 0.120) *p* = 0.494 | β = -0.232 (-3.214 ; 2.750) *p* = 0.879 | β = -2.078 (-4.133 ; -0.023) ***p* = 0.048** | β = -0.010 (-0.075 ; 0.056) *p* = 0.776 | β = -0.135 (-0.288 ; 0.018) ***p* = 0.083** | β = -1.864 (-4.221 ; 0.493) *p* = 0.121 | β = **-**1.007 (-1.929 ; -0.085) ***p* = 0.032** | β = -0.009 (-0.023 ; 0.005) *p* = 0.193 |
| **Total non-provitamin A** | OR: 1.025 (0.545 – 1.930) *p* = 0.938 | β = -0.011 (-0.199 ; 0.178) *p* = 0.911 | β = -0.383 (-0.679 ; -0.087) ***p =* 0.011** | β = -0.233 (-0.462 ; -0.005) ***p* = 0.045** | β = -2.843 (-6.534 ; 0.847) *p* = 0.131 | β = -3.633 (-3.175 ; -1.091) ***p* = 0.005** | β = -0.082 (-0.163 ; -0.000) ***p* = 0.049** | β = -0.007 (-0.196 ; 0.183) *p* = 0.945 | β = -0.485 (-3.407 ; 2.437) *p* = 0.745 | β = **-**0.049 (-1.193 ; 1.096) ***p* = 0.006** | β = -0.007 (-0.025 ; 0.010) *p* = 0.405 |
| **Total carotenoids** | OR: 0.616 (0.348 – 1.088) ***p* = 0.095** | β = -0.143 (-0.310 ; 0.024) ***p* = 0.094** | β = -0.375 (-0.636 ; -0.113) ***p =* 0.005** | β = -0.256 (-0.458 ; -0.054) ***p* = 0.013** | β = -1.122 (-4.393 ; 2.150) *p* = 0.501 | β = -2.836 (-5.090 ; -0.583) ***p* = 0.014** | β = -0.004 (-0.077 ; -0.068) *p* = 0.907 | β = -0.101 (-0.269 ; 0.066) *p* = 0.236 | β = -1.838 (-4.426 ; 0.750) *p* = 0.164 | β = **-**0.872 (-1.885 ; 0.140) ***p* = 0.091** | β = -0.011 (-0.027 ; 0.004) *p* = 0.149 |

ORISCAV: Observation of Cardiovascular Risk Factors.

Metabolic syndrome (MetS) was diagnosed with the NCEP-ATP III criteria.

siMetS score and siMetS risk score are continuous metabolic syndrome (MetS) scores used to quantify metabolic status and metabolic risk of cardio/cerebrovascular events, respectively.

Fruits and vegetables were not considered as confounders in this adjusted model (model 3). The confounders of model 3 are: age, sex, marital status, currently smoking status, job status, income, total energy intake and birth country.

*Regression analyses for siMetS risk score did not include sex and age as confounders, as they were included in the formula of this score.

All predictor variables were log-transformed before multivariable regression models.

BMI: Body mass index (kg/m^2^).

WHR: Waist-hip ratio.

Among anthropometric measurements including BMI and WHR, only waist circumference is considered as a component of MetS.

Data for logistic regression (only for MetS ATP III) are expressed as odds ratio (OR) with its 95% confidence interval (95% CI).

Data for linear regression are expressed as beta (β) regression coefficient with its 95% confidence interval (95% CI).

Total pro-vitamin A species corresponds to the sum of α-carotene, β-carotene and β-cryptoxanthin.

Epoxycarotenoids is the sum of neoxanthin and violaxanthin.

Phytoene+ phtofluene: the sum of phytoene and phtofluene represents colorless carotenoids.

Total non-provitamin A species corresponds to the sum of lycopene, lutein + zeaxanthin, astaxanthin, phytoene, phtofluene, neoxanthin and violaxanthin.

Total carotenoids corresponds to the sum of the total pro-vitamin A carotenoids and total non-provitamin A carotenoids.

Significant (*p* < 0.05) and tendency (*p* < 0.1) p-values are given in bold. *P*-values in blue ink indicate a tendency, i.e. *p*-value>0.05 but <0.1.

**Supplementary table 4.** Additional sensitivity analysis for models of logistic and linear regression for tomato-based carotenoids (lycopene, phytoene, phytofluene), taking into account potential confounding factors, i.e. processed tomato-based food items, associating them with metabolic syndrome (MetS), its scores, components and additional anthropometric measurements of Luxembourgish participants (n=1346) of the ORISCAV-2 study.

| **Carotenoid intake**  **(µg /day)** | **MetS (APT III)** | **siMetS score (metabolic status)** | **siMetS risk score (metabolic risk) *** | **Fasting blood glucose (FBG) (mmol /L)** | **Systolic blood pressure (SBP) (mmHg)** | **Diastolic blood pressure (DBP) (mmHg)** | **HDL-cholesterol (mmol /L)** | **Triglycerides (mmol /L)** | **Waist circumference (cm)** | **BMI (kg/m^2^)** | **WHR** |
| --- | --- | --- | --- | --- | --- | --- | --- | --- | --- | --- | --- |
| **Crude model** | | | | | | | | | | | |
| **Lycopene** | OR: 0.807 (0.590 – 1.104) *p* = 0.707 | β = 0.037 (-0.082 ; 0.157) *p* = 0.539 | β = -0.005 (-0.193 ; 0.182) *p* = 0.955 | β = -0.069 (-0.210 ; 0.072) *p* = 0.337 | β = -1.606 (-4.130 ; 0.918) *p* = 0.212 | β = -1.518 (-3.106 ; 0.071) ***p* = 0.061** | β = -0.036 (-0.090 ; 0.018) *p* = 0.190 | β = 0.061 (-0.055 ; 0.177) *p* = 0.303 | β = 0.061 (-1.923 ; 2.045) *p* = 0.952 | β = -0.072 (-0.771 ; 0.627) *p* = 0.840 | β = -0.003 (-0.018 ; 0.011) *p* = 0.638 |
| **Phytoene** | OR: 1.276 (0.859 – 1.894) *p* = 0.227 | β = -0.006 (-0.146 ; 0.134) *p* = 0.937 | β = -0.057 (-0.278 ; 0.164) *p* = 0.613 | β = -0.151 (-0.316 ; 0.015) ***p* = 0.074** | β = -2.881 (-5.844 ; 0.083) ***p* = 0.057** | β = -2.847 (-4.703 ; -0.992) ***p* = 0.003** | β = -0.014 (-0.077 ; 0.050) *p* = 0.676 | β = -0.074 (-0.210 ; 0.063) *p* = 0.289 | β = 0.356 (-1.974 ; 2.687) *p* = 0.764 | β = 0.287 (-0.533 ; 1.108) *p* = 0.492 | β = -0.005 (-0.022 ; 0.011) *p* = 0.529 |
| **Phytofluene** | OR: 1.198 (0.800– 1.795) *p* = 0.381 | β = -0.036 (-0.181 ; 0.108) *p* = 0.622 | β = -0.098 (-0.327 ; 0.130) *p* = 0.399 | β = -0.172 (-0.343 ; -0.001) ***p* = 0.048** | β = -3.662 (-6.721 ; -0.603) ***p* = 0.019** | β = -3.264 (-5.181 ; -1.347) ***p* = 0.001** | β = 0.001 (-0.065 ; 0.067) *p* = 0.975 | β = -0.100 (-0.240 ; 0.041) *p* = 0.165 | β = 0.243 (-2.167 ; 2.653) *p* = 0.843 | β = 0.305 (-0.546 ; 1.153) *p* = 0.480 | β = -0.007 (-0.025 ; 0.010) *p* = 0.394 |
| **Phytoene+Phytofluene** | OR: 1.264 (0.852 – 1.875) *p* = 0.245 | β = -0.008 (-0.148 ; 0.132) *p* = 0.910 | β = -0.059 (-0.279 ; 0.162) *p* = 0.603 | β = -0.150 (-0.315 ; 0.015) ***p* = 0.074** | β = -2.795 (-5.752 ; 0.161) ***p* = 0.064** | β = -2.817 (-4.669 ; -0.966) ***p* = 0.003** | β = -0.012 (-0.075 ; 0.051) *p* = 0.711 | β = -0.075 (-0.211 ; 0.061) *p* = 0.279 | β = 0.324 (-2.001 ; 2.648) *p* = 0.785 | β = 0.265 (-0.553 ; 1.083) *p* = 0.526 | β = -0.005 (-0.022 ; 0.011) *p* = 0.529 |
| **Model 2** | | | | | | | | | | | |
| **Lycopene** | OR: 0.876 (0.628 – 1.221) *p* = 0.434 | β = 0.068 (-0.044 ; 0.180) *p =* 0.237 | β = -0.005 (-0.193 ; 0.182) *p* = 0.955 | β = -0.030 (-0.164 ; 0.104) *p =* 0.660 | β = -0.581 (-2.750 ; 1.587) *p =* 0.599 | β = -1.153 (-2.663 ; 0.356) *p =* 0.134 | β = -0.034 (-0.082 ; 0.015) *p* = 0.174 | β = 0.076 (-0.037 ; 0.189) *p* = 0.186 | β = 0.629 (-1.098 ; 2.356) *p* = 0.475 | β = 0.064 (-0.611 ; 0.740) *p* = 0.852 | β = 0.001 (-0.009 ; 0.012) *p* = 0.789 |
| **Phytoene** | OR: 1.248 (0.803 – 1.939) *p* = 0.324 | β = 0.018 (-0.115 ; 0.150) *p =* 0.792 | β = -0.057 (-0.278 ; 0.164) *p* = 0.613 | β = -0.161 (-0.319 ; -0.003) ***p =* 0.046** | β = -2.577 (-5.144 ; -0.009) ***p =* 0.049** | β = -2.612 (-4.390 ; -0.835) ***p =* 0.004** | β = -0.080 (-0.137 ; -0.022) ***p* = 0.007** | β = -0.034 (-0.168 ; 0.099) *p* = 0.612 | β = 1.455 (-0.585 ; 3.495) *p* = 0.162 | β = 0.397 (-0.401 ; 1.195) *p* = 0.329 | β = 0.008 (-0.004 ; 0.020) *p* = 0.190 |
| **Phytofluene** | OR: 1.148 (0.731 – 1.804) *p* = 0.549 | β = -0.011 (-0.148 ; 0.127) *p =* 0.878 | β = -0.098 (-0.327 ; 0.130) *p* = 0.399 | β = -0.184 (-0.348 ; -0.020) ***p =* 0.028** | β = -3.340 (-5.993 ; -0.686) *p =* 0.807 | β = -3.011 (-4.851 ; -1.172) *p =* **0.001** | β = -0.073 (-0.132 ; -0.013) ***p* = 0.017** | β = -0.056 (-0.194 ; 0.082) *p* = 0.424 | β = 1.480 (-0.633 ; 3.593) *p* = 0.170 | β = 0.431 (-0.395 ; 1.258) *p* = 0.306 | β = 0.008 (-0.005 ; 0.020) *p* = 0.236 |
| **Phytoene+Phytofluene** | OR: 1.231 (0.793 – 1.910) *p* = 0.355 | β = 0.015 (-0.117 ; 0.147) *p =* 0.826 | β = -0.059 (-0.279 ; 0.162) *p* = 0.603 | β = -0.161 (-0.319 ; -0.003) ***p =* 0.045** | β = -2.504 (-5.065 ; 0.057) ***p =* 0.055** | β = -2.588 (-4.361 ; -0.815) ***p =* 0.004** | β = -0.077 (-0.135 ; -0.020) ***p* = 0.008** | β = -0.036 (-0.169 ; 0.097) *p* = 0.593 | β = 1.407 (-0.627 ; 3.442) *p* = 0.175 | β = 0.371 (-0.425 ; 1.167) *p* = 0.360 | β = 0.008 (-0.004 ; 0.020) *p* = 0.197 |
| **Model 3** | | | | | | | | | | | |
| **Lycopene** | OR: 0.887 (0.630 – 1.251) *p* = 0.495 | β = 0.071 (-0.040 ; 0.183) *p =* 0.210 | Β = -0.036 (-0.213 ; 0.141) *p =* 0.690 | β = -0.036 (-0.172 ; 0.100) *p* = 0.604 | β = -0.503 (-2.697 ; 1.692) *p* = 0.653 | β = -1.091 (-2.612 ; 0.430) *p* = 0.160 | β = -0.036 (-0.084 ; 0.013) *p* = 0.151 | β = 0.082 (-0.030 ; 0.195) *p* = 0.151 | β = 0.399 (-1.336 ; 2.133) *p* = 0.652 | β = 0.079 (-0.601 ; 0.759) *p* = 0.820 | β = 0.000 (-0.010 ; 0.011) *p* = 0.926 |
| **Phytoene** | OR: 1.230 (0.770 – 1.966) *p* = 0.387 | β = 0.021 (-0.116 ; 0.157) *p =* 0.765 | β = -0.222 (-0.435 ; -0.009) ***p =* 0.041** | β = -0.190 (-0.355 ; -0.025) ***p* = 0.024** | β = -2.548 (-5.229 ; 0.133) ***p* = 0.062** | β = -2.817 (-4.665 ; -0.969) ***p =* 0.003** | β = -0.086 (-0.145 ; -0.027) ***p* = 0.004** | β = -0.018 (-0.155 ; 0.120) *p* = 0.802 | β = 0.615 (-1.500 ; 2.731) *p* = 0.568 | β = 0.294 (-0.536 ; 1.123) *p* = 0.487 | β = 0.006 (-0.006 ; 0.019) *p* = 0.312 |
| **Phytofluene** | OR: 1.116 (0.686 – 1.815) *p* = 0.658 | β = -0.011 (-0.152 ; 0.131) *p =* 0.884 | β = -0.236 (-0.483 ; -0.042) ***p =* 0.020** | β = -0.216 (-0.387 ; -0.045) ***p* = 0.013** | β = -3.340 (-6.115 ; -0.565) ***p* = 0.018** | β = -3.290 (-5.204 ; -1.375) ***p =* 0.001** | β = -0.078 (-0.139 ; -0.017) ***p* = 0.012** | β = -0.040 (-0.182 ; 0.103) *p* = 0.583 | β = 0.564 (-1.631 ; 2.759) *p* = 0.614 | β = 0.304 (-0.556 ; 1.165) *p* = 0.488 | β = 0.006 (-0.007 ; 0.019) *p* = 0.380 |
| **Phytoene+Phytofluene** | OR: 1.212 (0.760 – 1.936) *p* = 0.419 | β = 0.018 (-0.118 ; 0.154) *p =* 0.799 | β = -0.224 (-0.437 ; -0.012) ***p =* 0.039** | β = -0.190 (-0.354 ; -0.025) ***p =* 0.024** | β = -2.469 (-5.143 ; 0.205) ***p =* 0.070** | β = -2.785 (-4.628 ; -0.942) ***p =* 0.003** | β = -0.084 (-0.142 ; -0.025) ***p* = 0.005** | β = -0.020 (-0.156 ; 01172) *p* = 0.780 | β = 0.571 (-1.539 ; 2.681) *p* = 0.596 | β = 0.268 (-0.559 ; 1.096) *p* = 0.525 | β = 0.006 (-0.006 ; 0.019) *p* = 0.322 |

ORISCAV: Observation of Cardiovascular Risk Factors.

Metabolic syndrome (MetS) was diagnosed with the NCEP-ATP III criteria.

siMetS score and siMetS risk score are continuous metabolic syndrome (MetS) scores used to quantify metabolic status and metabolic risk of cardio/cerebrovascular events, respectively.

Model 1: unadjusted model; Model 2: adjusted model for sex and age; Model 3: adjusted for age, sex, marital status, currently smoking status, job status, income, total energy intake and birth country.

*Regression analyses for siMetS risk score did not include sex and age as confounders, as they were included in the formula of this score.

Sensitivity of the 3 models was increased, considering the following processed tomato-based food items as confounders: pizza, burger, ketchup, and lasagna with meat and tomato sauce.

All predictor variables were log-transformed before multivariable regression models.

BMI: Body mass index (kg/m^2^).

WHR: Waist-hip ratio.

Among anthropometric measurements including BMI and WHR, only waist circumference is considered as a component of MetS.

Data for logistic regression are expressed as odds ratio (OR) with its 95% confidence interval (95% CI).

Data for linear regression are expressed as beta (β) regression coefficient with its 95% confidence interval (95% CI).

Total pro-vitamin A species corresponds to the sum of α-carotene, β-carotene and β-cryptoxanthin.

Epoxycarotenoids is the sum of neoxanthin and violaxanthin.

Phytoene+ phtofluene: the sum of phytoene and phtofluene represents colorless carotenoids.

Total non-provitamin A species corresponds to the sum of lycopene, lutein + zeaxanthin, astaxanthin, phytoene, phtofluene, neoxanthin and violaxanthin.

Total carotenoids corresponds to the sum of the total pro-vitamin A carotenoids and total non-provitamin A carotenoids.

Significant (*p* < 0.05) and tendency (*p* < 0.1) *p*-values are given in bold. *P*-values in blue ink indicate a tendency, i.e. *p*-value>0.05 but <0.1.
